# No-flip ShangRing circumcision in 10-12 year old boys: Results from randomized clinical trials in Kenya

**DOI:** 10.1371/journal.pone.0233150

**Published:** 2020-05-22

**Authors:** Omar Al Hussein Alawamlh, Quentin D. Awori, Mark A. Barone, Soo-Jeong Kim, Marc Goldstein, Philip S. Li, Richard K. Lee

**Affiliations:** 1 Department of Urology, James Buchanan Brady Foundation, Center for Male Reproductive Medicine and Microsurgery, Weill Cornell College of Cornell University, New York, NY, United States of America; 2 EngenderHealth, Nairobi, Kenya; 3 EngenderHealth, Washington, DC, NY, United States of America; IAVI, UNITED STATES

## Abstract

**Background:**

Attention has recently turned toward the use of device-assisted male circumcision to help scale up male circumcision services in sub-Saharan Africa, with increasing emphasis on younger age groups. We assessed the use of the ShangRing for circumcising the subset of boys aged 10 to 12 years who were enrolled in two randomized clinical trials in Kenya.

**Methods:**

We performed a sub-analysis of outcomes in 197 boys aged 10 to 12 years; a subset who were enrolled in two randomized clinical trials to assess the use of the no-flip ShangRing circumcision technique in men and boys. One trial assessed spontaneous detachment vs. planned removal of the ShangRing 7 days post-circumcision. The second trial compared the use of topical vs. injectable anesthesia with ShangRing circumcision. Aside from baseline characteristics, data was collected and analyzed for each trial separately.

**Results:**

All participants were successfully circumcised. Duration of circumcision, participants requiring a dorsal slit, rate of adverse events, time to complete wound healing, and participant satisfaction were similar between the two groups in each trial. Mean time required for spontaneous ShangRing detachment was 14.82±3.76 days. Topical anesthesia showed a significantly lower mean pain score at the time of application (0.64±1.71 vs. 1.55± 2.21, p = 0.03) as well as postoperatively (0.54±0.88 vs. 1.72±1.56, p<0.0001). Median dwell time of the topical anesthetic was 43 (IQR: 35.5–60) minutes, while the median time it took the injectable anesthetic to take effect was 2.04 (IQR: 1.72–3.09) minutes.

**Conclusion:**

No-flip ShangRing circumcision had a positive safety profile among young adolescent boys, specifically ages 10–12 years. The use of spontaneous device detachment and topical anesthesia with the procedure have shown promising outcomes in this age group. This may have the potential to further increase the acceptability of ShangRing circumcision, and therefore accelerate the scle up of male circumcision services in sub-Saharan Africa.

**Trial registration:**

ClinicalTrials.gov registration # NCT02390310.

## Introduction

Voluntary medical male circumcision (VMMC) has been shown to reduce HIV incidence in men by 50–60% in three randomized trials. Male circumcision (MC) also reduces the risk of human papilloma virus (HPV), herpes simplex virus type 2 (HSV-2) in men; trichomonas vaginalis and bacterial vaginosis infections in their female partners; genital ulcer disease in both sexes; as well as cervical and penile cancers [1–4]. Further, MC provides the great benefits of protecting against urinary tract infections, phimosis, and balanitis in boys [5, 6]. These results have led the World Health Organization (WHO) and the Joint United Nations Program on HIV/AIDS (UNAIDS) to recommend the inclusion of VMMC in countries with low MC rates and high heterosexual HIV transmission as part of their HIV prevention strategies [7]. Given this notion, 14 priority sub-Saharan countries have begun the scale-up of VMMC efforts, however only 44% of the goal was achieved by the year 2016 [8]. Part of this shortcoming is attributed to the scarcity of trained providers and resources in the aforementioned region.

With the new WHO target of 27 million circumcisions by 2021, device-assisted VMMC has been proposed to simplify the procedure, reduce the burden on healthcare providers, and enable safe task-shifting from physicians to non-physician medical providers, thus increasing the availability of MC services in Africa [8]. The ShangRing is a single-use, disposable device which consists of two concentric plastic rings; a lockable outer ring and an inner ring which is lined with a silicon band. It is one of two WHO-prequalified MC devices that has demonstrated excellent safety and acceptability results compared to conventional circumcision in previous studies conducted in Africa [9, 10].

Recently, results from men and boys aged 10 to 54 years who were enrolled in two randomized clinical trials of no-flip ShangRing circumcision in Kenya have been published [11, 12]. In addition to assessing the safety, acceptability, and efficacy of the ShangRing, the first trial aimed to assess spontaneous device detachment as part of postoperative care management, and the second trial sought to evaluate the use of topical anesthesia with the procedure. Here, we report on results from a sub-analysis of 10 to 12 year old boys from the aforementioned cohort who took part in the trials. The importance of this age group resides in its inclusion in the new priority age group (10 to 29 years) of the World Health Organization’s revised 2021 VMMC target, to accelerate the scale up of MC services in sub-Saharan Africa [13]. Additionally, the no-flip ShangRing technique has fairly recently received WHO prequalification for use in 10 to12 year olds [14]; therefore, we aim to provide further evidence of the safety and efficacy of the procedure with spontaneous device detachment and the use of topical anesthesia among boys of this age group.

## Methods

We performed a sub-analysis of outcomes in 10 to 12 year old males from results of two randomized controlled clinical trials of no-flip ShangRing circumcision in men and boys aged 10 to 54 years [11, 12]. Both trials were conducted at two sites; the Homa Bay County Teaching and Referral Hospital, Homa Bay, Kenya and Vipingo Health Center in Kilifi County, Kenya. 197 boys aged 10 to 12 years were recruited for both trials. The first trial assessed the use of spontaneous ShangRing detachment, which allows the ring to naturally fall on its own, *vs*. planned ring removal at day 7 following no-flip ShangRing circumcision. The second trial assessed the use of topical *vs*. injectable anesthesia for no-flip ShangRing circumcision.

Parents or legally accepted representatives (LARs) were first asked to sign an informed consent form to document the voluntary decision of their child’s participation in the study. Assent was also obtained from the boys who understood study procedures. All 10 to 12 year old boys in each trial were then screened and examined to determine eligibility. Inclusion criteria for both trials included: no previous circumcision, participant is in good general health and free of genital ulcerations or signs of infection. Boys with active genital infection, previous circumcision, an anatomic abnormality or another condition that contraindicated elective surgery under local anesthesia (e.g. bleeding diathesis, lidocaine allergy) were excluded from enrollment.

Participant randomization in both trials was stratified by age with blocks of varying sizes. 50% of all recruited participants in each trial were aged 10–15 years and the other 50% were above the age of 15. The random allocation of participants in both trials was conducted with the use of a text-message service (Sealed Envelope Ltd., London, UK). A computer-generated allocation sequence was performed by a researcher and then uploaded onto the service. The staff at the study sites randomized participants prior to circumcision, however, blinding to allocation was not feasible due to the limited staff and overall nature of the study.

1 Spontaneous ShangRing detachment *vs*. planned ring removal on day 7 following no-flip ShangRing circumcision

**Fig 1:**
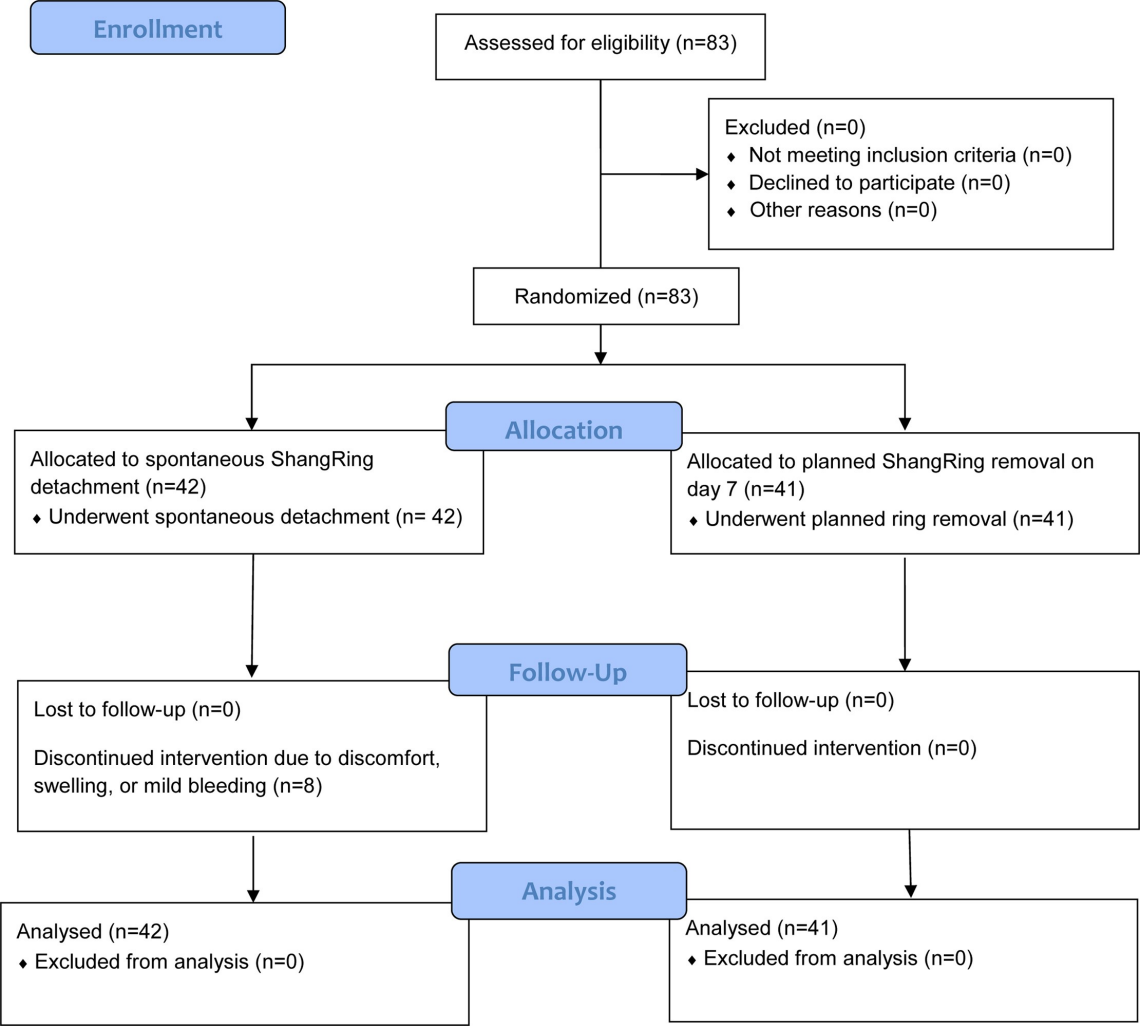
CONSORT flow diagram: Spontaneous ShangRing detachment vs. planned ring removal on day 7 following no-flip ShangRing circumcision.

**Fig 2:**
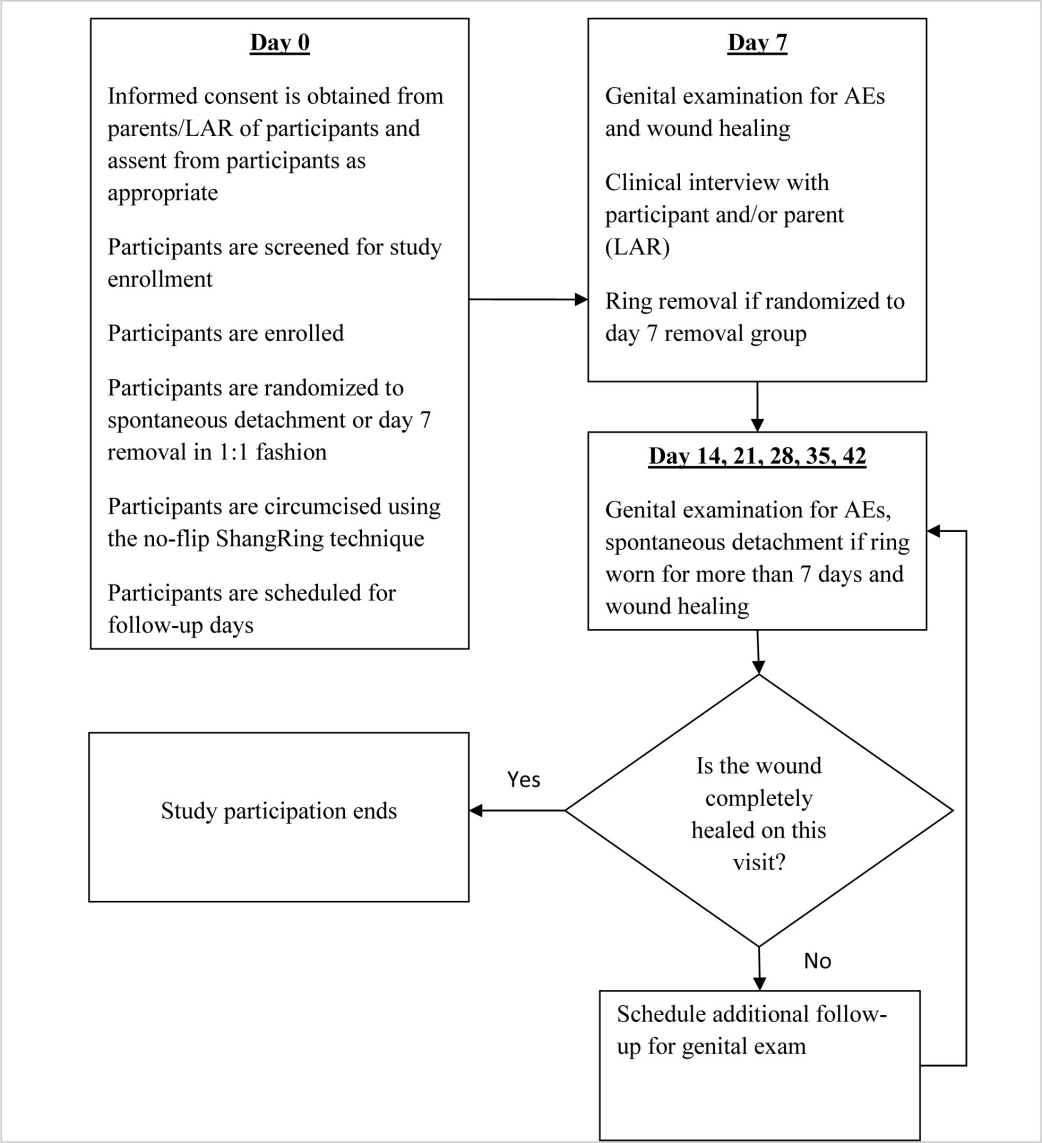
Spontaneous ShangRing detachment *vs*. planned ring removal on day 7 following no-flip ShangRing circumcision, procedure flow.

A total of 83 boys aged 10 to 12 years were first recruited to undergo ShangRing circumcision using the no-flip technique [15–17]. Participants were randomized 1:1 to either spontaneous detachment of the ShangRing following circumcision vs. ShangRing removal on day 7 postoperatively (Fig 1). Recruitment began on May 8^th^ of 2015 with the last participant randomized on September 16^th^ of the same year. Baseline demographic data were gathered, and a clinical exam was performed. The appropriate size of the ShangRing was determined using the ShangRing measuring strip. Local anesthesia with dorsal penile nerve and ring blocks was administered using 1% lidocaine for both groups after they were prepped and draped in standard surgical fashion. No-flip ShangRing circumcision was then carried out by trained providers by inserting the inner plastic ring of the ShangRing device under the foreskin with secure clamping of the outer ring to provide hemostatic occlusion. The foreskin distal to the device was then excised. A dorsal slit was performed to facilitate the insertion of the inner ring if phimosis was present. Peri-procedural parameters were recorded, including procedural time as well as pain score using the Wong-Baker FACES Visual Analogue Pain Scale (VAS). Follow-up visits were conducted on days 7, 14, 21, 28, 35 and 42 after circumcision and were completed on October 28^th^, 2015. Additional visits were scheduled for those who did not achieve full wound healing by day 42. Patients were encouraged to return at any time if they experienced an adverse event (AE), excessive pain, or other problem. At each visit, genital exams and interviews were conducted. Photographs were taken to document complete wound healing and AEs. Fig 2 outlines the flow of procedures during the trial.

2 Topical *vs*. injectable anesthesia for no-flip ShangRing circumcision

**Fig 3:**
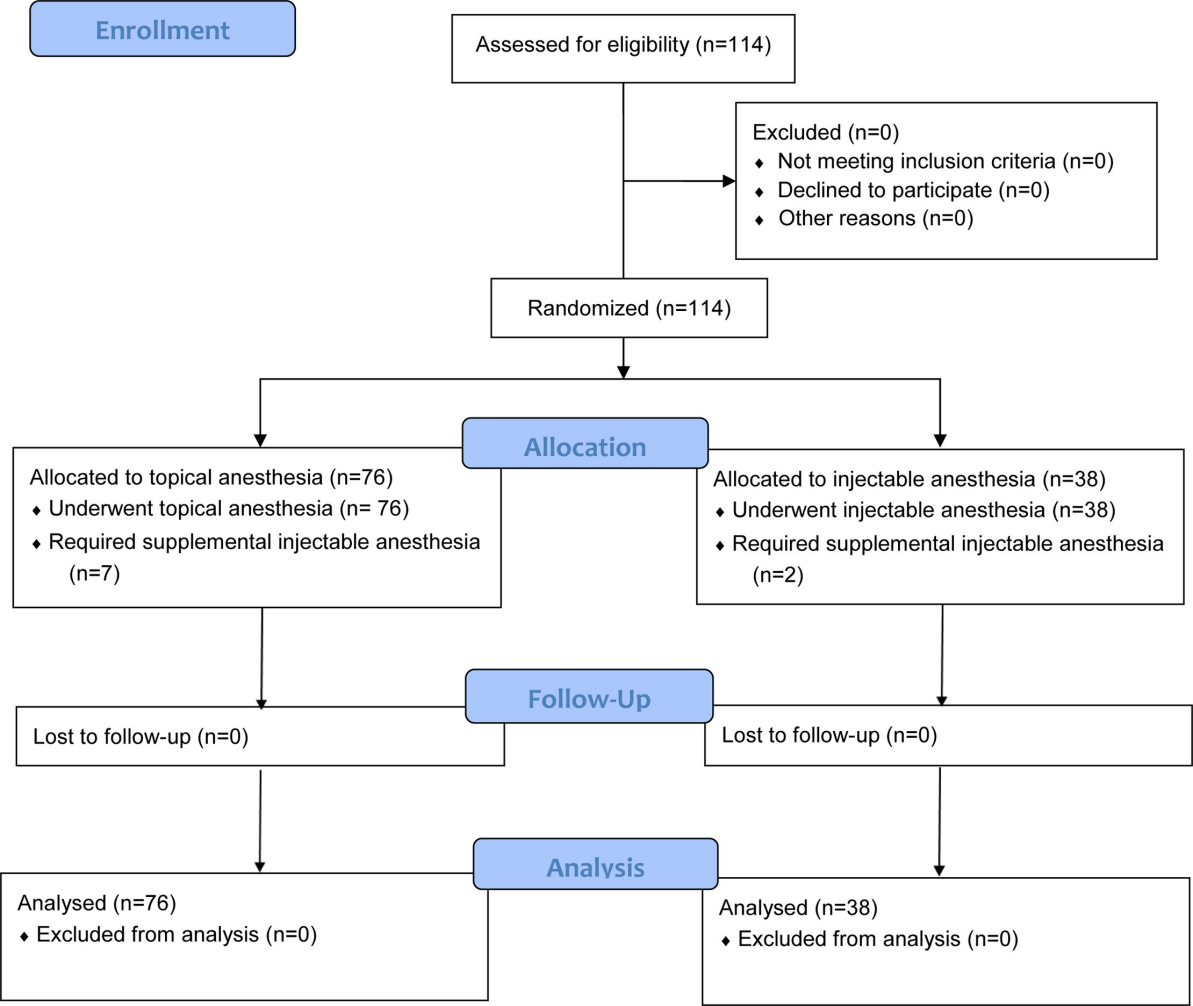
CONSORT flow diagram: Topical *vs*. injectable anesthesia for no-flip ShangRing circumcision.

114 boys aged 10 to 12 years were next recruited to undergo ShangRing circumcision using the no-flip technique. These participants were randomized in 2:1 fashion to undergo circumcision after topical anesthesia with 2.5% lidocaine/2.5% prilocaine *vs*. injectable anesthesia with 1% lidocaine (Fig 3). The 2:1 randomization scheme was used since all participants in the first trial had undergone injectable anesthesia and pain scores were reported for the entire cohort [11]; therefore, it was unnecessary to have a 1:1 randomization. However, given the subjectivity when reporting pain scores, it was critical to randomize participants in this trial.

**Fig 4:**
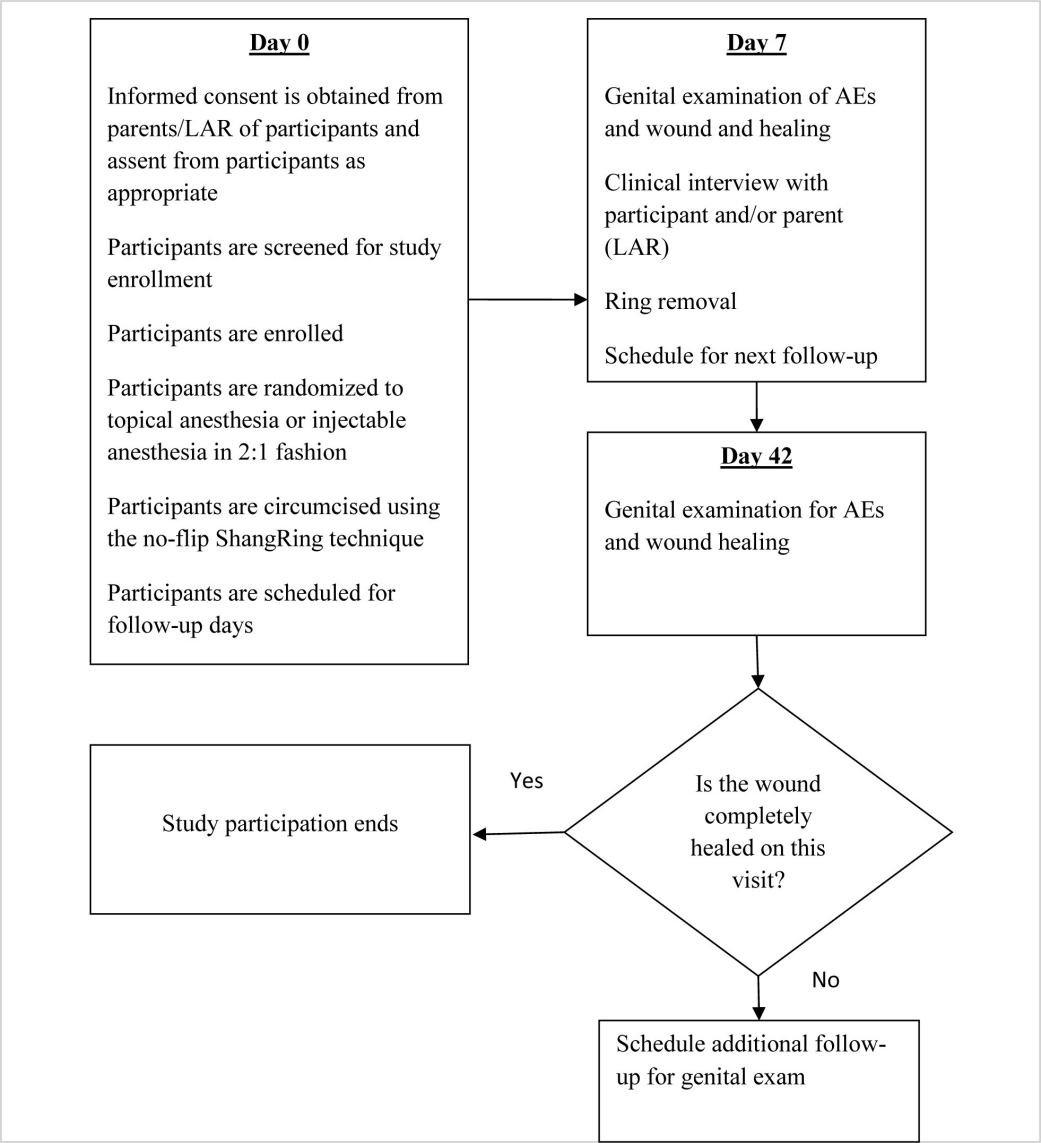
Topical *vs*. injectable anesthesia for no-flip ShangRing circumcision, procedure flow.

Participant recruitment began on November 11^th^, 2015 and the last participant randomized in the trial was on June 7^th^ of 2016. In the topical group, the anesthetic was applied from the tip of the penis to the base, including the inner and outer surfaces of the foreskin, and then allowed to dwell from 25 minutes up to 70 minutes. Dwell time was defined as the time between the application of the anesthetic cream and start of the procedure. Twenty minutes into the dwell time, the effect of the topical anesthetic was initially assessed and then every 5 to 10 minutes thereafter. Of note, total anesthetic dwell time was primarily a function of provider availability and not just time of onset for anesthesia, and so it was not an accurate measure of how long it took the topical anesthetic to take effect. Injectable anesthesia was applied in similar fashion to the first trial, i.e. through dorsal penile nerve and ring blocks. Baseline demographic data were gathered prior to circumcision and a clinical exam was performed. Participants were then surgically prepped, ShangRing size determined, and no-flip ShangRing circumcision performed by trained providers as in the first trial. A dorsal slit was performed in case of phimosis. Peri-procedural parameters were obtained, including VAS pain scores, anesthetic dwell time, and total procedural time. Follow-up visits were conducted only on days 7 and 42 post-circumcision. The last follow-up visit was completed on July 19^th^, 2016. All participants had the device removed on the day 7 visit. Additional visits were scheduled for those who did not achieve full healing by day 42. Participants were encouraged to return at any time if they experienced an AE, excessive pain, or other problem. At each visit, genital exams and interviews were conducted. Photographs were taken to document complete wound healing and AEs. Fig 4 outlines the flow of procedures during the trial.

### Sample size and interim analysis

The sample sizes for the trials were determined in accordance with the recommendations from the WHO for the clinical evaluation of male circumcision devices [18]. This includes ruling out a 5% adverse event rate. For the first trial, the sample size of each group provided 80% power to detect an increase of 7% of adverse events with the use of no-flip ShangRing circumcision with either spontaneous device detachment or planned removal 7 days post-operatively, using a one-sided test and alpha of 0.05. This is based on historic adverse event data of previous ShangRing studies that have utilized the standard flip technique with planned device removal 7 days post-circumcision [11]. In the second trial, the sample size provided 100% power to detect a mean difference of 1 on the 0–10 pain scale with a standard deviation of 1.8, using a two-sided test at alpha of 0.05. This is based on prior data from a study of the ShangRing that had detected a mean between-group difference of 1 point on the 11-point pain scale [10, 12].

For other data collected, sample sizes for both trials provided at least 80% power using a two-sided test and alpha of 0.05. A single planned interim analysis was conducted by a three-member Data and Safety Monitoring Board (DSMB) for each trial; this was after conducting 40 no-flip ShangRing procedures with the device removed at 7 days post-operatively in the first trial, and after a total of 80 no-flip ShangRing procedures with the use of topical anesthesia in the second trial. The DSMB were required to recommend the continuation or halting of participant recruitment upon reviewing pertinent data relevant to the benefit or harm of the procedure or with evidence of futility. Since the analysis was planned and intended to monitor safety, no data was gathered for the purposes of sample size determination or adjustment and hence did not impact final sample sizes in the trials. Following the DSMB’s review of the interim analysis, both trials were recommended to continue.

### Data collection and statistical analysis

Data on participant eligibility, procedural success, duration of circumcision, rate of AEs, time to complete healing, need for a dorsal slit, pain scores, time of ring removal/detachment, as well as participant satisfaction with the appearance of the healed penis were collected. AEs were pre-specified according to the WHO Technical Advisory Group on Innovations in Male Circumcision and classified as related or unrelated to circumcision [19, 20]. Moderate and severe AEs were photographed for subsequent review. Complete wound healing was defined as the absence of a scab with a completely epithelialized and dry skin surface based on clinical assessment. Wound healing was assessed at each site by the clinician examining the participant. Time to wound healing was calculated and compared between the different participant groups. χ^2^, *t*-test, and Mann-Whitney *U* test were used for statistical analysis (SPSS v15, IBM Corporation, Armonk, NY). Alpha level for significance was set at 5%. Missing data was not imputed.

### Ethical approval

The study and informed consent forms were reviewed and approved by the Kenya Medical Research Institute Scientific and Ethics Review Unit (KEMRI SERU) and the Weill Cornell Medical College Institutional Review Board (WCMC IRB). Regulatory approval was also obtained from the Kenya Pharmacy and Poisons Board. The study was registered with ClinicalTrials.gov at NCT02390310.

## Results

A total of 197 participants aged 10 to 12 years were enrolled. All were found to be suitable and eligible for no-flip ShangRing circumcision after meeting inclusion criteria. 83 and 114 (100%) boys from the first and second trials, respectively, were successfully circumcised using the no-flip ShangRing technique by trained physicians and health care providers. Selected baseline characteristics of participants and reasons for circumcision were combined as demonstrated in Table 1.

**Table 1:** Baseline characteristics of participants and reasons for circumcision from both trials.

**Number of participants**	197
Ethnicity*	
• Luo, n (%)	126 (64.6)
• Giriama, n (%)	40 (20.5)
• Chonyi, n (%)	15 (7.7)
• Other, n (%)	14 (7.2)
Religion*	
• Pentecostal, n (%)	62 (31.8)
• Seventh day Adventist, n (%)	44 (22.6)
• Roman Catholic, n (%)	32 (16.4)
• No religion, n (%)	23 (11.8)
• Anglican, n (%)	17 (8.7)
• Muslim, n (%)	13 (6.8)
• Other	4 (2.0)
Reason for circumcision*	
• Hygiene, n (%)	106 (54.4)
• Social/Religious, n (%)	49 (25.1)
• HIV protection, n (%)	39 (20.0)
• Other, n (%)	1 (0.5)

*Data missing from 2 participant for ethnicity, from 2 participant for religion, and from 2 participant for reason for circumcision.

1 Spontaneous ShangRing detachment *vs*. planned ring removal on day 7 following no-flip ShangRing circumcision

42 boys were randomized to spontaneous ShangRing detachment *vs*. 41 to planned ring removal on day 7 post-circumcision. Median duration of circumcision was equivalent for both groups [6.28 (IQR: 4.93–8.18) minutes *vs*. 6.52 (IQR: 5.43–8.30) minutes, p = 0.57]. The number of participants requiring dorsal slits due to phimosis to facilitate inner ring insertion was also equivalent between the two groups (27 vs. 27, p = 0.88). All boys in the day 7 removal group had successful removal of the ring as scheduled. In the spontaneous detachment group, 8 (19%) participants had requested ring removal prior to ring detachment. 7 (87.5%) of the 8 participants who requested ring removal were due to complaints of either discomfort or swelling, while it was a provider’s decision to have the ring removed for the last participant due to mild bleeding. The mean day of spontaneous detachment was 14.82±3.76 days.

Wound healing was not affected by the use of spontaneous detachment *vs*. planned ring removal, as all participants from both groups were completely healed by the last follow-up visit on day 42. 2 participants from each arm experienced procedure-related AEs (4.9% *vs*. 4.8%, p = 0.98). All AEs were edema of moderate grade. These were managed conservatively using non-steroidal anti-inflammatory medication (paracetamol and ibuprofen) and antibiotics (flucloxacillin) without any sequelae. 98% of spontaneous detachment participants and 100% of day 7 removal participants were satisfied with the appearance of the healed penis after complete wound healing was achieved (Table 2).

**Table 2:** Spontaneous ShangRing detachment *vs*. planned ring removal on day 7 following no-flip ShangRing circumcision, participant data by randomization group.

Randomization group	Spontaneous detachment	Day 7 removal	*p*
• Participant eligible for ShangRing circumcision, n (%)	42 (100)	41 (100)	1.00
• Procedure successfully completed, n (%)	42 (100)	41 (100)	1.00
• Median duration of circumcision in minutes (IQR)	6.28 (4.93–8.18)	6.52 (5.43–8.30)*	0.57
• Participants who required a dorsal slit, n (%)	27 (64.3)	27 (65.9)	0.88
• Adverse events, n (%)	2 (4.9)	2 (4.8)	0.98
• Complete wound healing by day 42, n (%)	42 (100)	41 (100)	1.00
• Ring was removed before intended day or spontaneous detachment, n (%)	8 (19)	0 (0)	0.01
• Participant satisfied with appearance of healed penis, n (%)	38 (98)**	38 (100)***	0.55

*Data missing from 3 participants in the day 7 removal group for duration of circumcision.

**Data missing from 3 participants in the spontaneous detachment group for participant satisfied with appearance of healed penis.

***Data missing from 2 participants in the day 7 removal group for participant satisfied with appearance of healed penis.

2 Topical *vs*. injectable anesthesia for no-flip ShangRing circumcision

76 participants were randomized to topical anesthesia vs. 38 to injectable anesthesia. Similar to the first trial, median duration of circumcision [4.31 (IQR: 3.48–5.40) minutes vs. 4.48 (IQR: 3.55–6.20) minutes, p = 0.44] and the number of participants requiring a dorsal slit (16 vs. 11, p = 0.37) due to phimosis were equivalent between the two groups. There were no reported AEs. Pain scores at the time of anesthesia application was significantly higher for injectable anesthesia with a mean score of 1.55±2.21, while participants who received topical anesthesia reported a score of 0.64±1.71 during the application of the anesthetic cream (p = 0.03). Although the effect of the anesthetic cream was assessed at intervals, the total dwell time was recorded and had a median of 43 (IQR: 35.5–60). The time it took the injectable anesthetic to take effect was 2.04 (IQR: 1.72–3.09) minutes.

8 (10.6%) participants of the topical anesthesia group required additional injectable anesthesia prior to the start of the procedure vs. 2 (5.6%) participants from the injectable group who also required supplemental anesthesia (p = 0.55). Pain control during the procedure was comparable among the two groups with a mean pain score of 0.28±0.84 in the topical group vs. 0.11±0.51 in the injectable group (p = 0.18). Topical anesthesia achieved significantly better pain control postoperatively when participants were assessed 20 minutes after undergoing circumcision; mean pain score of 0.54±0.88 vs. 1.72±1.56 (p<0.0001). All (100%) participants from both groups achieved complete wound healing by day 42, and the vast majority were satisfied with the appearance of the healed penis at that time (94.7% of participants in the topical group vs. 92.1% of injectable group, p = 0.58, Table 3).

**Table 3:** Topical *vs*. injectable anesthesia for no-flip ShangRing circumcision, participant data by randomization group.

Randomization group	Topical anesthesia	Injectable anesthesia	*p*
• Participant eligible for ShangRing circumcision, n (%)	76 (100)	38 (100)	1.00
• Procedure successfully completed, n (%)	76 (100)	38 (100)	1.00
• Median duration of circumcision in minutes (IQR)	4.31 (3.48–5.40)*	4.48 (3.55–6.20)	0.44
• Participants who required a dorsal slit, n (%)	16 (21.1)*	11 (28.9)	0.30
• Adverse events, n (%)	0 (0)	0 (0)	-
• Mean pain score at the time the anesthetic was applied (SD)	0.64 (1.71)*	1.55 (2.21)	0.03
• Mean pain score during the procedure (SD)	0.28 (0.84)*	0.11 (0.51)	0.18
• Mean pain score 20 minutes post-operatively (SD)	0.54 (0.88)**	1.72 (1.56)***	<0.0001
• Participant required additional anesthetic supplementation, n (%)	8 (10.6)*	2 (5.6)	0.55
• Complete wound healing by day 42, n (%)	76 (100)	38 (100)	1.00
• Participant satisfied with the appearance of the healed penis, n (%)	72 (94.7)**	35 (92.1)	0.58

*Data missing from 1 participant in topical anesthesia group for median duration of circumcision, participants who required a dorsal slit, mean pain score at the time the anesthetic was applied, mean pain score during the procedure, and participant required additional anesthetic supplementation.

**Data missing from 4 participants in topical anesthesia group for mean pain score 20 minutes post-operatively.

***Data missing from 2 participant in injectable anesthesia group for mean pain score 20 minutes post-operatively.

^Data missing from 4 participants in topical anesthesia group for participant satisfied with appearance of healed penis.

## Discussion

This study is the first to exclusively report on the use of no-flip ShangRing circumcision in young adolescent boys aged 10 to 12 years, a part of the WHO targeted VMMC priority age group for HIV prevention [13].

No-flip ShangRing circumcision was safe in young adolescents, with a low rate of adverse events. Of note, the procedure was successfully carried out in all participants of this age group despite the considerable number of participants who possessed phimosis. The presence of phimosis has posed a significant issue with the PrePex device (Circ MedTech, Hod Hasharon, Israel), another WHO prequalified MC device that is now no longer being produced. In their reports, the WHO had indicated that Prepex circumcision was not feasible with foreskin conditions, including phimosis, thus limiting its use in a good proportion of adolescents [19, 21, 22]. The time needed to perform the procedure in 10 to 12 year old boys was found to be similar to that of the rest of the cohort in the respective trials [11, 12]. This was also similar to the duration of circumcision with the standard ShangRing technique, which has been reported in previous trials to be significantly less than conventional circumcision techniques [9, 10, 20]. Participant satisfaction with cosmesis was excellent in both trials regardless of the method of device detachment or type of local anesthetic.

Spontaneous detachment of the device was a viable management technique following ShangRing circumcision in 10 to 12 year olds. The ShangRing successfully fell on its own in 81% of patients with a mean of approximately 14 days in this age group. The majority of those who did not wait for spontaneous detachment requested ring removal because of discomfort or swelling. Results from the entire cohort of the first trial had shown that 72.4% of participants from all age groups awaited the device to detach on its own with a median time to detachment of 14 days. Pain and discomfort were the primary reasons for requested removal [11]. None of the boys experienced device displacements or malfunctions. The use of spontaneous detachment did not lead to an increased rate of AEs nor did it affect the rate of wound healing, which was in line with older age groups [11]. The low rate of total AEs and the absence of any severe or serious AE was consistent with previously reported rates of ShangRing circumcision [23–25]. It is important to point out however, that since edema was the only procedure related AE encountered among the boys in this trial, we recommend that providers pay close attention when choosing the ShangRing size. In a cohort of 104 boys aged 6 to 14 years, Fang, et al. noted that an increase in the difference between ShangRing size and glans size was associated with an increased risk of edema [24]. We also recommend that in the event that the measurement falls between two sizes, the smaller size should be chosen to minimize the risk of postoperative edema.

The use of topical anesthesia with ShangRing circumcision was also viable in the young adolescent age group. Although injectable anesthesia is considered the gold standard for circumcision, the use of a needle is considered painful, especially when compared with the application of a cream. Our results were consistent with this notion, with significantly increased pain scores seen at the time of anesthetic application in the injectable group. Topical anesthetic provided equivalent pain control during the time of circumcision and in fact provided superior pain control following circumcision. Results from the entire cohort of this trial were similar to what was seen in this age group, with respect to pain control among the two study groups at the different time points in which pain scores were captured [12]. From a programmatic perspective, the use of a topical anesthetic might make circumcision more appealing to target populations due to less pain at the time of application and to the superior analgesia seen afterwards. It is interesting to note that although dwell time in this study was longer than the latency time required for injectable anesthesia, dwell time did not reflect the true latency period required for the topical agent to become effective; rather, it was primarily a measure of provider availability as staff were busy carrying out circumcisions for multiple participants on the same day. As a rough measure, however, one can safely assume that the latency time for a topical anesthetic would likely be less than 30 minutes. Also interesting was the fact that postoperative analgesia seemed to be prolonged in those receiving topical anesthetic. The tradeoff of longer time of onset for extended duration of effect is intriguing.

This study had several limitations. First, the sample sizes of 10 to 12 year old boys in the sub-analysis of the two trials were relatively small. As such, safety and efficacy results, while promising, may be difficult to generalize. Next, the latency time required for topical anesthesia did not in reality serve as a function of time required for anesthesia onset only; it included delays for when the study staff were busy with other clinical activities. Thus, dwell time estimate likely represents an overestimate of true anesthesia latency time. Time to complete wound healing may have also been overestimated, as follow-up visits were required only at seven-day intervals in the first trial and on days 7 and 42 post-circumcision in the second trial, where participants could have achieved complete wound healing in between scheduled follow-up visits. Despite the inability to accurately report anesthetic dwell time and time to complete wound healing, nonetheless, our results are likely an actual representation of programmatic settings for VMMC services in areas which lack trained healthcare providers.

## Conclusion

No-flip ShangRing circumcision had a positive safety profile among young adolescent boys, specifically ages 10–12 years. The use of spontaneous device detachment and topical anesthesia with the procedure have shown promising outcomes in this age group. This may have the potential to further increase the acceptability of ShangRing circumcision, and therefore accelerate the scale up of male circumcision services in sub-Saharan Africa.

## Supporting information

S1 File CONSORT checklist. (DOCX)

S2 File Study protocol. (PDF)

S1 Data (XLSX)

S2 Data (XLSX)

S3 Data (XLSX)
