# Supplementary material for: No-flip ShangRing circumcision in 10-12 year old boys: Results from randomized clinical trials in Kenya
Source: PLoS One. 2020 May 22;15(5):e0233150. doi: 10.1371/journal.pone.0233150 (PMC7244118; doi:10.1371/journal.pone.0233150)
Supplement: S2 File — (PDF) [file pone.0233150.s002.pdf]

PROTOCOL

Simplifying the Shang Ring Technique for Circumcision of Men and Boys

Sponsors: **EngenderHealth**
440 Ninth Avenue
New York, NY 10001-1620, USA

Weill Cornell Medical College
525 E. 68th Street
New York, NY 10065, USA

Funded By: **The Bill & Melinda Gates Foundation**

Principal Investigator **Mark A. Barone, DVM, MS**
EngenderHealth, NY, USA

Protocol Executive Committee:
Quentin D. Awori, **MBChB,** EngenderHealth Nairobi, Kenya
Raymond Otieno, **MBChB, MMED (Surg)**, Homa Bay County Hospital, Homa Bay, Kenya
Mark A. Barone, **DVM, MS,** EngenderHealth, NY, USA
Philip S. Li, MD, Weill Cornell Medical College, NY, USA
Richard Lee, MD, Weil Cornell Medical College, NY, USA
Marc Goldstein, **MD**, Weil Cornell Medical College, NY, USA

Investigator Team:
John Masasabi Wekesa, MBChB, MMed(Surg), Ministry of Medical Services, Nairobi, Kenya
Athanusias Ochieng, MBChB, National AIDS & STI Control Programme, Nairobi, Kenya
George Githuka, MBChB, National AIDS & STI Control Programme, Kenya
Hashim Suleiman, MBChB, Kilifi district hospital, Kilifi County
Judy Kinya, **HSM(MSc), CMS(Dip.)** Vipingo Health Center, Kilifi County, Kenya
Jared Moguche, **MBChB**, EngenderHealth, Nairobi, Kenya
Betty Chirchir, MPH,Epid.(Cert), BDS, Bon Santé Consulting, Nairobi, Kenya

Site Principal Investigators:
Raymond Otieno, MBChB, MMED (Surg)
Homa Bay Country Hospital

Judy Kinya, HSM (MSc), CMS (Dip.)
Vipingo Health Center

Monitors:

EngenderHealth
440 Ninth Avenue
New York, NY 10001-1620, USA

Weill Cornell Medical College
525 E. 68th Street
New York, NY 10065, USA

Study Sites:

Homa Bay County Hospital, Homa Bay, Kenya
Vipingo Health Center, Kilifi County, Kenya

Simplifying the Shang Ring Technique for Circumcision of Men and Boys

Date __29__ / __04__ / __2015__

SITE PRINCIPAL INVESTIGATOR'S STATEMENT

I, the Site Principal Investigator, agree to conduct this study in full accordance with the provisions of this protocol and will comply with all requirements regarding the obligations of clinical investigators as fully outlined in the International Conference on Harmonization (Section E6(R1) Good Clinical Practice) and in the Statement of Investigator (Form US FDA 1572), which I have also signed. I agree to maintain all study documentation until EngenderHealth advises that it is no longer necessary. I also agree to publish or present data only upon review and after discussion with EngenderHealth and Cornell.

I have read and understand the information in this protocol, including the potential risks and side effects of the product under investigation, and will ensure that all associates, colleagues, and employees assisting in the conduct of the study are informed about the obligations incurred by their contribution to the study.

______________________________ ________________________

Signature of Investigator **Date (dd/mm/yyyy)**

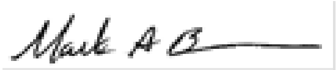

29/04/2015

Signature of Principal Investigator **Date (dd/mm/yyyy)**
(on behalf of Protocol Executive Committee)

ACRONYMNS

**AE**	Adverse Experience or Adverse Event
**CE**	European Conformity
**CFR**	Code of Federal Regulations (US)
**CMS**	Clinical Medicine and Surgery
**CRF**	Case Report Form
**eCRF**	Electronic Case Report Form
**DSMB**	Data and Safety Monitoring Board
**EH**	EngenderHealth
**GCP**	Good Clinical Practice
**HIV**	Human Immunodeficiency Virus
**HPV**	Human Papilloma Virus
**HSV-2**	Human Herpes Virus – Type 2
**ICH**	International Conference on Harmonization
**IRB**	Institutional Review Board
**KEMRI**	Kenya Medical Research Institute
**LAR**	Legally acceptable representative
**MC**	Male Circumcision
**M&E**	Monitoring and Evaluation
**MOH**	Ministry of Health
**PEC**	Protocol Executive Committee
**PI**	Principal Investigator
**RCT**	Randomized Controlled Trial
**RE**	Research Ethics
**SAE**	Serious Adverse Event
**SNNDA**	Wuhu SNNDA Medical Treatment Appliance Technology Co., Ltd
**SOP**	Standard Operating Procedure
**SHE**	Social Harm Event
**SR**	Shang Ring
**STI**	Sexually Transmitted Infection
**US FDA**	United States Food and Drug Administration
**VMMC**	Voluntary Medical Male Circumcision
**VCT**	Voluntary Counseling and Testing
**WCMC**	Weill Cornell Medical College
**WHO**	World Health Organization

Table of Contents

ACRONYMNS 4
SUMMARY 7
1.0 INTRODUCTION 8
1.1 Background 8
1.2 Rationale 9
1.3 The Shang Ring Male Circumcision Device & Previous Studies 10
1.3.1 Shang Ring Clinical Studies in China 11
1.3.2 Shang Ring Clinical Studies in Africa 11
2.0 STUDY OBJECTIVES 15
2.1 Phase I 15
2.1.1 Primary Objectives 15
2.1.2 Secondary Objectives 15
2.2 Phase II 15
2.2.1 Primary Objectives 15
2.2.2 Secondary Objectives 15
3.0 ENDPOINTS 16
3.1 Phase I 16
3.1.1 Primary Endpoints 16
3.1.2 Secondary Endpoints 16
3.2 Phase II 16
3.2.1 Primary Endpoints 16
3.2.2 Secondary Endpoints 16
4.0 STUDY DESIGN 17
5.0 STUDY SITE 21
6.0 STUDY PARTICIPANTS 21
6.1 Population Base 21
6.2 Inclusion Criteria 21
6.3 Exclusion Criteria 22
6.4 Client Withdrawal/Discontinuation 22
7.0 STUDY PRODUCT 22
7.1 The Shang Ring Device 22
7.2 The Shang Ring Circumcision Procedure and Removal 24
7.3 Labeling and Packaging of Shang Ring Supplies 24
7.4 Device Accountability 25
7.4.1 Device Shipment and Handling 25
7.4.2 Product Tracking 25
7.4.3 Product Tracking and Disposition after Use 25
7.5 EMLA Cream 26
8.0 STUDY VISITS & PROCEDURES 26
8.1 Recruitment 26
8.2 Screening, Enrollment, and Circumcision Visit 27
8.3 Post-Op Follow-Up Visits 29
8.4 Interim and Unscheduled Visits 31
8.5 Summary of Activities at each Study Visit 32
8.6 Participant Compensation 33
9.0 STATISTICAL CONSIDERATIONS 33
9.1 Study Size Justification 33
9.2 Analysis Plan Summary 33
9.3 Interim Analysis 34

10.0 MANAGEMENT OF INTERCURRENT EVENTS 35
10.1 Loss to Follow-Up 35
10.2 Adverse Events 36
10.3 Serious Adverse Events (SAE) 36
10.4 Social Harm Events 38
11.0 PROTOCOL NONCOMPLIANCE 38
12.0 PROTECTION OF HUMAN SUBJECTS 39
12.1 Institutional Review 39
12.2 Informed Consent and Assent 39
12.3 Risks and Benefits 40
12.3.1 Risks of the Shang Ring Male Circumcision Procedure 40
12.3.2 Benefits of the Shang Ring Male Circumcision Procedure 41
12.4 Participant Confidentiality 42
13.0 DATA MANAGEMENT 42
13.1 Overview 42
13.2 Record Retention and Data Storage 43
13.3 Data Collection, Transmission and Storage 43
14.0 QUALITY CONTROL AND QUALITY ASSURANCE 44
15.0 SUMMARY OF STUDY MONITORING 45
16.0 PUBLICATION AND PRESENTATION OF RESULTS 45
17.0 ACKNOWLEDGMENTS 46
18.0 APPENDIXES 47
Appendix I: Shang Ring circumcision procedure 47
Appendix II: Shang Ring removal procedure 48
Appendix III: European CE Mark (2008) 49
Appendix IV: EN ISO certificate on November 26, 2007 52
19.0 REFERENCES 54

SUMMARY

Design:

The study, to be conducted in two phases, will examine procedural and clinical outcomes, as well as participant and provider acceptability, of adaptations of the Shang Ring technique for male circumcision that would simplify its use.

- Phase I will explore use of the no-flip technique with historical data from standard Shang Ring circumcisions in Africa used as the comparison group. Men will be randomized to Shang Ring removal at 7 days vs. being followed to observe for spontaneous detachment,
- Phase II will be a randomized trial comparing use of topical vs. injectable anesthesia.

Population:

The study population will include Kenyan men and boys ages 10 years and older seeking circumcision at a study sits. A total of 230 participants will be recruited into Phase I and 345 into Phase II. Because of the possibility that boys and men could have different outcomes, we will recruit participants equally in two age groups in both phases; 10-15 and >15 years old.

Duration:

Approximately 8 months for completion through follow-up of the two phases of the study (exact timing will depend on recruitment rate). In the first phase, participants shall be followed-up at 7, 14, 21, 28, 35, and 42 days post circumcision, although study participation will end once complete wound healing has been documented. In the second phase, participants shall be followed up at 7 and 42 days post-circumcision. In both phases men will be followed beyond 42 days if necessary to document complete wound healing.

Primary Objectives & Endpoints:

Phase I: Assess safety of the no-flip technique for Shang Ring circumcision by determining the rate of moderate and severe adverse events (AE) following use of the no-flip technique.

Phase II: Assess pain experienced during the Shang Ring circumcision procedure with topical vs. injectable anesthesia. The outcome metric would be maximum pain experienced during the Shang Ring circumcision, assessed using the visual analogue scale (VAS) score, reported immediately after completion of the procedure.

Sites:

Homa Bay County Hospital, Homa Bay County, Kenya
Vipingo Health Center, Kilifi County, Kenya

1.0 INTRODUCTION

1.1 Background

Male circumcision has been proven to dramatically reduce female-to-male HIV transmission. In 2006, the results of three separate randomized control trials (RCTs) conducted in sub-Saharan Africa established the benefit of voluntary medical male circumcision (VMCC) in resulting in a 60% reduction in men's risk of acquisition of heterosexual HIV (Auvert et al., 2005; Bailey et al., 2007; Gray et al., 2007). More recently, the protective effect of male circumcision against HIV infection has been shown to last for at least four to five years (Bailey et al., 2010; Gray et al., 2012). In South Africa, roll-out of male circumcision services in Orange Farm township has significantly reduced HIV incidence among circumcised men (Lissouba et al., 2011).

In addition, male circumcision provides protection against transmission of other sexually transmitted infections, making men less susceptible to acquiring or transmitting genital ulcer disease, trichomonas and gonorrhea (Marrazzo and Cates, 2011, Auvert et al., 2009, Gray et al., 2009, Halperin and Bailey, 1999, Nasio et al., 1996). Data also show that male circumcision provides significant protection against both human papillomavirus (HPV) and herpes simplex virus type 2 (HSV-2), reducing the risk of infection by about one-third for both viruses, (Auvert et al., 2009, Backes et al., 2012, Serwadda et al., 2010, Wilson et al., 2013) in addition to reducing the risk of cervical cancer in female sex partners of circumcised men (Albero et al., 2012, Davis et al., 2013, Wawer et al., 2011).

Today, MC is the only method of HIV prevention shown to be consistently effective in reducing HIV risk. The World Health Organization (WHO) and the Joint United Nations Program on HIV/AIDS (UNAIDS) recommend that countries with low MC rates, high HIV prevalence, and predominantly heterosexual epidemics include VMMC as part of their comprehensive HIV prevention strategies (WHO and UNAIDS 2007). Governments such as Kenya's have rapidly reassessed their HIV prevention strategies and policies, and have added VMMC services as an ambitious nationwide goal (Kenya MOPHS, 2009). Over a dozen countries in sub-Saharan have done the same (WHO and UNAIDS, 2011).

Rapid scale-up of VMMC in sub-Saharan Africa could drastically reduce HIV incidence. Word of the benefits of VMMC has spread across communities, and demand for the procedure among boys and men has increased exponentially—even among those for whom circumcision is not traditionally practiced as part of a cultural rite of passage. Widespread deployment of VMMC services in the developing world has been slow, however, hampered by both human and material resource constraints. As of the end of 2012, only 15% of the 20 million target by the year 2015 has been achieved in sub-Saharan Africa as a whole (WHO 2013a). This calls for more intense program implementation and service delivery, in particular the development of male circumcision (MC) procedures that can be performed safely and effectively by less skilled providers.

One of the major obstacles to expanding VMMC is the number of skilled health workers and the amount of time and resources that are required to provide large numbers of circumcisions using the conventional surgical techniques currently recommended by WHO (WHO, UNAIDS, and JHPIEGO, 2008; Auvert et al., 2008). The relative technical difficulty of these techniques, coupled with the scarcity of trained providers in Sub-

Saharan Africa and other resource-poor regions, has limited the availability of VMMC services. Surgery typically takes 20-40 minutes to complete, requires suturing, and poses risks related to bleeding and infection.

A tidal shift towards device-assisted VMMC is hence needed if the invaluable benefits of MC in achieving a public health impact are to be realized. Devices for MC hold the promise of a simpler and faster procedure that could facilitate more rapid scale-up of MC services. One single-use disposable device, the Shang Ring, requires less surgical skill and can be safely used by non-physicians, allowing for task-shifting. Shang Ring procedures do not require suturing, a time-consuming and skill-requiring step in surgical MC.

1.2 Rationale

The Shang Ring (Fig. 1) is a novel medical device with the potential to revolutionize how MC services are offered in countries hardest hit by the HIV epidemic. Recent research by EngenderHealth, Weill Cornell Medical College, and others has shown that the Shang Ring method requires less time, is simpler and easier to teach and perform, and is preferred by providers over conventional surgical techniques (Barone et al., 2011, Barone et al., 2012, Barone et al., 2013, Kigozi et al., 2013, Sokal et al., 2014,). In two recent studies, our RCT in Kenya and Zambia (Sokal et al., 2014) and an acceptability study conducted in Uganda (Kigozi et al., 2013), men preferred the Shang Ring to conventional circumcision. To continue exploring innovative approaches and techniques that could make Shang Ring circumcisions even faster and simpler, we propose research to evaluate two new innovations for the technique: 1) the use of a no-flip technique and 2) the use of topical anesthesia in place of injectable local anesthesia.

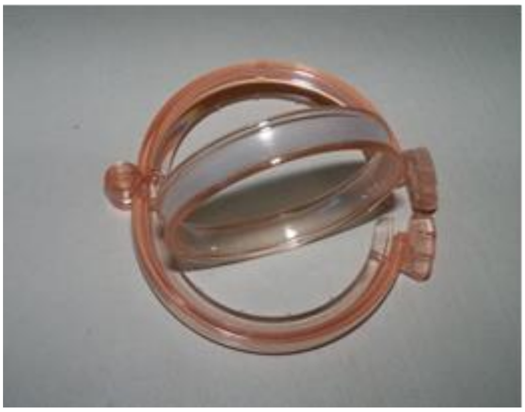

Fig 1. The Shang Ring male circumcision device

The proposed research builds on work currently ongoing in Kunming and Wuhu, China, where encouraging initial results have been seen (Yan et al., 2010, Yang et al., in press 2014). With standard Shang Ring MC, the inner ring is placed over the glans penis and the foreskin is flipped, or everted, over the inner ring before placing and closing the outer ring, sandwiching the foreskin between the rings (see Appendix I). With the no-flip technique, the inner ring is placed inside the foreskin, at the level of the glans penis. The outer ring is positioned on the outside of the foreskin over the inner ring and closed to sandwich the foreskin between the rings (see Appendix I). The removal technique is the same for both the standard and no-flip Shang Ring techniques (see Appendix II)

Advantages of the no-flip technique over the standard technique include: 1) shorter procedure time, 2) easier circumcision procedure and device removal, and 3) simpler training. Providers in Kenya and Zambia have reported that the most difficult part of the procedure was everting the foreskin, a step which is eliminated in the no-flip technique. Preliminary data from China, where more than 150 no-flip Shang Ring circumcisions on men 18 years old and above have been conducted (as well as more than 650 no-flip procedures in children), suggest that the no-flip method is faster and easier and has no serious problems during or post-circumcision (Yan et al., 2010). Spontaneous

detachment of the Shang Ring may be more likely following the no-flip technique compared to following the standard Shang Ring technique. A video of the no-flip technique can be watched at: http://www.youtube.com/watch?v=MjJlQgU4HDw.

Another potential innovation that could significantly improve the treatment paradigm for Shang Ring MC is the replacement of injectable local anesthetics with topical agents. Injected anesthesia, the current standard for Shang Ring circumcision, requires a skilled provider and is the most painful part of the procedure. Some men who fear needles may avoid circumcision, and use of a topical anesthetic would potentially improve acceptability of MC. Additionally, hypodermic needles can pose a risk to the provider. Reuse of needles and syringes is a potential source of infectious transmission, and safe disposal of needles and syringes can be problematic. Therefore, identifying an effective topical agent would significantly improve the treatment paradigm for Shang Ring MC and alleviate needle disposal concerns. In addition to being easier, topical anesthesia will likely be better tolerated when the faster no-flip technique is used.

Topical anesthesia is used with another male circumcision device, PrePex (Bitega et al., 2011, Mutabazi et al., 2012, Galukande et al., 2014). Although the mechanism of action of the PrePex Device is different, it does provide a theoretical basis for use of topical anesthesia with MC devices. Indeed, recently another new MC device, named the Unicirc was introduced with a mechanism of action is similar to the Shang Ring using topical anesthesia (see video at https://www.youtube.com/watch?v=e3i1MIhV1ZU). Additionally, there are some preliminary promising results from use of topical anesthesia in men and boys in China (personal communication). With application of topical anesthetic cream to the mucosal layer of the foreskin and dwell times of 15-45 minutes, adequate anesthesia to carry out circumcision is achieved. While data from China are limited and preliminary, they suggest that topical anesthesia for Shang Ring MC is worth pursuing more systematically.

Thus, in moving towards accelerated use of Shang Ring without injected anesthesia, this two-phase study will first evaluate use of the no-flip technique in an African population of males 10 years of age and older, and then in the second phase, explore topical anesthesia in a randomized study of topical vs. injected anesthesia, using the no-flip technique if it is found safe and acceptable (otherwise, the standard Shang Ring technique would be used in Phase II). The two phases may overlap as described under section 4.0 on page 17.

1.3 The Shang Ring Male Circumcision Device & Previous Studies

Commercially available in China since 2005, the Shang Ring is a novel disposable MC device that consists of a set of two concentric plastic rings (Fig. 1). It is available in 32 sizes, ranging from 9 to 42 mm in diameter, for use in neonates to adults. Hemostasis is provided by compression of the foreskin between the locking rings, which minimizes bleeding that occurs with the conventional surgical approaches and obviates the need for sutures that are a necessary and time consuming part of the conventional techniques. It significantly simplifies the circumcision procedure, which makes it extremely promising for use in low-resource settings by a variety of health workers, in particular non-physician clinicians (nurses and clinical officers). The Shang Ring allows a circumcision to be performed with only a few simple steps within an average time of 5 minutes or less (excluding anesthesia) versus the conventional surgical procedures that average 20-40 minutes.

1.3.1 Shang Ring Clinical Studies in China

Results from Chinese studies have demonstrated that the Shang Ring is both safe and easy to use. The first report of the Shang Ring in the literature was from a study conducted by Peng et al. (2008) who reported low rates of complications among 1,200 Shang Ring circumcisions. Similarly low complication rates were seen in a series of 328 men circumcised with the Shang Ring in another Chinese study by Cheng et al. (2009). A third study by Li et al. (2010) comparing Shang Ring circumcision with the dorsal slit technique found significantly fewer complications, lower pain levels during and after the procedure, and higher participant satisfaction among participants circumcised with the Shang Ring. Average Shang Ring circumcision times (excluding administration of local anesthesia), were reported in the Chinese studies to be between 3 to 5 minutes with patient satisfaction rates over 98% (Peng et al., 2008, Cheng et al., 2009, Li et al., 2010, Yang et al., in press 2014).

1.3.2 Shang Ring Clinical Studies in Africa

Table 1 provides a summary of Shang Ring studies conducted in Africa (for a detailed review, see Barone et al., 2014).

Table 1. Shang Ring clinical studies in Africa.

**Study**<br>**[type of study]**	**Location**	**Number and type of participants**	**Type of provider**
**Safety Study in Kenya**<br>[case series]<br>(Barone et al., 2011)	Homa Bay District Hospital,<br>Kenya	40 healthy HIV-negative men	Physicians and nurses experienced in conventional surgical circumcision and trained in China to conduct Shang Ring circumcisions
**Spontaneous Detachment Study in Kenya**<br>[randomized comparative trial]<br>(Barone et al., 2012)	Homa Bay District Hospital,<br>Kenya	50 healthy HIV-negative men	Physicians and nurses experienced in conventional surgical circumcision and trained in China to conduct Shang Ring circumcisions
**Randomized Comparison with Conventional Surgical Circumcision in Kenya and Zambia** [randomized comparative trial] (Sokal et al., 2014)	Homa Bay District Hospital, Homa Bay Kenya and University Teaching Hospital, Lusaka, Zambia:	400 healthy HIV-negative men (200 allocated to Shang Ring circumcision and 200 to conventional surgical circumcision)	Physicians, nurses and clinical officers experienced in conventional surgical circumcision and trained in China to conduct Shang Ring circumcisions
**Field Studies in Kenya and Zambia**<br>[field studies]<br>(Barone et al., 2013)	Seven sites in Homa Bay, Kenya and three sites in Lusaka, Zambia	1211 healthy HIV-negative and HIV positive men	Physicians, nurses and clinical officers experienced in conventional surgical circumcision who were either trained in China or trained locally by those trained in China to conduct Shang Ring circumcisions
**Acceptability and Safety In Uganda**<br>[field study]<br>(Kigozi et al., 2013)	Rakai Health Sciences Programme, Rakai District, Uganda	621 healthy HIV-negative men (508 chose Shang Ring circumcision and 113 chose conventional surgical circumcision)	Clinical officers experienced in conventional surgical circumcision and trained in Kenya to conduct Shang Ring circumcisions

Study<br>[type of study]	Location	Number and type of participants	Type of provider
**Safety study with boys in Kenya**<br>[case series]	Homa Bay District Hospital,<br>Kenya	80 healthy boy between 3 months and <18 years old	Physicians and nurses experienced in Shang Ring circumcision in adults, trained to conduct Shang Ring circumcisions in children in China or Kenya

Adapted from WHO Technical Advisory Group on Innovations in Male Circumcision: Evaluation of Two Adult Devices. Meeting report: Published in August 2013. Geneva, Switzerland. ISBN 978 92 4 150563 5.

Between October to December 2009, a pilot study was conducted at the Homa Bay District Hospital that sought to evaluate the safety, efficacy and acceptability of the Shang ring in the Africa population (Barone et al., 2011). This was the first use of the device outside China, and 40 adult clients were enrolled. The results showed that:

1. The Shang Ring was efficient, with short procedure times, and suitable for MC in adult men in Kenya.
2. No severe or moderate AEs were seen in this study.
3. Additionally, the device was well-tolerated, with men reporting minimal disruption to daily life and low pain scores during erections (3.5±2.3, 0=no pain and 10=worst pain possible). All men reported they were very satisfied, happy with the appearance of their circumcised penis and would recommend the Shang Ring to others .
4. This confirmed that the Shang Ring had the potential to greatly facilitate the safe and effective scale-up of MC services, especially by trained non-physicians, in settings with high HIV prevalence and low MC use.

Subsequently, between October 2010 to January 2011, we conducted a “Time to Removal” study that evaluated whether the device would spontaneously detach if removal was delayed for longer than the currently recommended time of 7 days and if wound healing was affected by different removal times (Barone et al., 2012). Fifty participants were randomized to removal of the Shang Ring at three different time points: 7, 14 and 21 days post-circumcision. The results showed that:

1. There are no serious consequences if men do not return at 7 days for removal as detachments occurred without significant problems among those who had the ring in place for more than 7 days. Complete detachment of the device occurred in 22 (66.7%) men who wore it more than 7 days.
2. Seven men (14.0%) with partial detachments requested removal 8-14 days post-circumcision due to pain or discomfort caused by the partially detached ring contacting the healing wound. Healing occurred uneventfully in these men.
3. Ring removal at later times following adult MC does not alter the time to complete healing; cumulative probabilities of complete healing were similar across groups.

These results confirm those of the pilot study that the Shang Ring is safe, acceptable and easy to use according to the label instructions of removal at day 7 post- circumcision.

In March to Aug 2011, we conducted an RCT that compared Shang Ring MC to conventional surgical circumcision techniques (Sokal et al., 2014). This study was conducted in Homa Bay, Kenya and Lusaka, Zambia. A total of 400 men 18 and older

were enrolled 200 each undergoing either Shang Ring or conventional MC. The RCT concluded that:

1. Pain scores and adverse event rates (15/197, 7.6% vs. 10/201, 5.0% in the Shang Ring and conventional surgery groups, respectively) were similar between the two groups
2. Significantly more men were "very satisfied" with appearance of their penis after Shang Ring MC (95.7% vs. 85.9% in Kenya and 96.8% vs. 71.3% in Zambia)
3. The Shang Ring technique took one-third the time of conventional techniques (7 vs. 20 minutes)
4. Complete wound healing with the Shang Ring was slower by about 5 days compared to conventional technique (mean 44.1±12.6 vs. 38.9±12.6 days)
5. Providers preferred the Shang Ring over conventional techniques

As safety profiles of the two groups were similar, all of the MC providers preferred the Shang Ring technique, and the study participants preferred the Shang Ring's cosmetic results, it appears that the Shang Ring is a good alternative to conventional surgery for VMMC scale-up.

A comparative study of the Shang Ring to the dorsal slit method was conducted in Rakai, Uganda. HIV-negative men 18 years and older seeking male circumcision were provided detailed information on the two MC methods. Those interested in participating in the study were asked to choose their preferred procedure. A total of 621 men were enrolled in the study, with the majority (508/621, 81.8%) choosing the Shang Ring over the dorsal slit procedure (113/621, 18.2%). Reasons men gave for choosing the Shang Ring included shorter procedure time and the belief that it was safer and would be a less painful procedure. Men who chose dorsal slit circumcision did so because it represented the standard procedure. They believed it was safer; and it did not require a removal procedure. Other results showed that:

1. In four (0.8%) patients, the Shang Ring procedure was not able to be performed, and circumcision needed to be completed with traditional suture-based technique. The ring slipped off after the foreskin was excised in three cases, and there was damage to the foreskin after the Shang Ring had been placed in the last case. All of the device failures occurred early in the study and were attributed by study investigators to provider inexperience
2. Shang Ring circumcisions took approximately 1/3 the time of conventional ones (6 vs. 17 minutes)
3. There was no difference in the rate of moderate and severe adverse events between the groups (5/500, 1.0% in the Shang Ring group vs. 1/117, 0.9% in the dorsal slit group)
4. Significantly more men in the dorsal slit group (111/113, 100%) demonstrated complete wound healing at 4 weeks follow-up compared to the Shang Ring group (408/486, 84.0%, ($p<0.001$)
5. There was no difference between the two groups in the proportion of men "satisfied" or "very satisfied" with the circumcision procedure they chose (99.1% in the Shang Ring group vs. 100% in the dorsal slit group)

In June, 2012, we completed a large prospective field study of use of the Shang Ring in routine services in Kenya and Zambia. The study was conducted at seven sites in Homa

Bay District, Nyanza Province, Kenya and three sites in Lusaka, Zambia. Providers at eight of the ten sites had no prior experience with the Shang Ring; they were trained to conduct Shang Ring circumcisions prior to the start of the study. We enrolled over 1,200 men 18 years and older, approximately 600 each in Kenya and Zambia between February and May, 2012. We evaluated the safety of the technique when used in a routine service setting, including the identification of rare or unexpected device-related events. We also collected data on client acceptability and provider preferences.

The field study demonstrated that:

1. The adverse event rate was low (2.0%) and well within the reported range following conventional MC reported in the literature
2. The majority (95%) of men reported returning to normal activities by the third day after circumcision
3. Healing was similar among HIV-infected and uninfected men, with 90.5% and 91.3% completely healed, respectively, by the 35-42 day visit
4. Most men (94.7%) were very satisfied with the appearance of their circumcised penis. Almost all men (99.8%) would recommend circumcision to a friend or family member and 98.8% would specifically recommend a Shang Ring circumcision
5. Of the 21 providers, 19 (90.5%) preferred the Shang Ring over conventional surgery

The Shang Ring has a good safety profile in routine service delivery settings and is preferred by most clients and providers, making it a good option for scaling up adult VMMC in Africa.

Most recently, we completed a pilot study of use of the Shang Ring in children in Kenya. This was the first use of the device in children of all ages, including infants, outside of China. A study of the Shang Ring in adolescent boys 16-17 years old is ongoing in Uganda. Published studies from China found the Shang Ring easy and simple to use with shorter procedure times, fewer complications and better cosmetic results compared to conventional MC techniques for children (Yan et al., 2010; Pan et al., 2013). In our pilot study in Kenya, a total of 80 boys from four age groups were enrolled: <1 (n=6), 1-5 (n=30), 6-12 (n=23), and 13-17 (n=21) years old. Participants were circumcised using the Shang Ring no-flip technique and rings were removed 5-7 days post-circumcision. Results showed that:

1. Circumcision and device removal times were quick (mean circumcision and device removal times 7.5±3.1 and 4.5±2.2 minutes, respectively)
2. There were no severe AEs. Four moderate AEs were noted and were managed successfully with conservative management
3. Mean healing time was 29.9 ± 7.3 days
4. Most patients or their parents/guardians (94.7%) were very satisfied with cosmesis and would recommend Shang Ring circumcision to others (95.0%)

The results of this pilot study suggest that the Shang Ring for male circumcision in boys is safe, effective and acceptable. Larger prospective trials are required to further validate the use of the Shang Ring in the pediatric population. In the most recent pilot study, we successfully used the no-flip technique in 30 boys between the ages of 10 and

17 years, the first use of the this technique outside of China. In the study described in this protocol, we will study the no-flip technique in a larger sample of men and boys 10 years of age and older.

2.0 STUDY OBJECTIVES

2.1 Phase I

2.1.1 Primary Objectives

- Assess safety of the no-flip technique for Shang Ring circumcision in males aged 10 years and older

2.1.2 Secondary Objectives

- Assess ease of use of the Shang Ring with the no-flip technique
- Evaluate satisfaction of the no-flip Shang Ring among study participants
- Evaluate satisfaction of using the no-flip Shang Ring technique among MC providers
- Determine the occurrence and safety of spontaneous detachment
- Evaluate acceptability of wearing the Shang Ring for more than 7 days following circumcision

2.2 Phase II

2.2.1 Primary Objectives

- Compare pain experienced during the Shang Ring circumcision procedure with topical vs. injectable anesthesia. EMLA Cream (lidocaine 2.5% and prilocaine 2.5%) will be the topical anesthetic used and the injectable anesthetic will be 1% lidocaine, the standard currently used in the Kenya National VMMC program.

2.2.2 Secondary Objectives

- Compare safety of topical vs. injectable anesthesia for local anesthesia during Shang Ring circumcision
- Compare ease of use of topical vs. injectable anesthesia for local anesthesia during Shang Ring circumcision
- Explore service optimization for use of topical anesthesia in conjunction with Shang Ring circumcision
- Compare duration of post-procedural analgesic effects between the topical vs. injectable anesthesia groups
- Compare satisfaction with the topical vs. injectable anesthetic among study participants
- Compare satisfaction with the topical vs. injectable anesthetic among circumcision providers

3.0 ENDPOINTS

3.1 Phase I

3.1.1 Primary Endpoints

- We will address the primary objective by evaluating the rate of moderate and severe adverse events[i] (AEs) following Shang Ring MC using the no-flip technique based on clinical exam findings. Other safety endpoints will include documentation of time to complete clinical wound healing,[ii] device-related malfunctions and occurrences of device displacement.

3.1.2 Secondary Endpoints

We will address the secondary objectives by evaluating the following endpoints:

- To evaluate ease of use with the no-flip technique we will document MC procedure and device removal times and problems encountered during MC and removal procedures;
- To evaluate satisfaction with the no-flip technique and the acceptability of wearing the Shang Ring for more than 7 days following circumcision among study participants and as appropriate their parents/LAR, we will interview participants/parents/LARs to document acceptability and satisfaction with the Shang Ring circumcision, procedure and post-procedure pain, and time to return to normal activity; and
- To evaluate satisfaction among MC providers we will interview providers to document experiences with use of the no-flip technique, problems encountered during MC procedures, and preference of MC technique.
- To determine the occurrence and safety of spontaneous detachment we will gather data on timing of spontaneous detachment and document problems/AEs and time course of complete clinical wound healing at clinical examination, as well as problems/complaints reported during interviews of participants (and as appropriate parents/LARs) among those participants wearing the Shang Ring for more than 7 days after circumcision

3.2 Phase II

3.2.1 Primary Endpoints

- The outcome metric for the primary outcome will be maximum pain reported to have been experienced by participants during the Shang Ring circumcision, assessed using the visual analogue scale (VAS), reported immediately after completion of the Shang Ring circumcision procedure

3.2.2 Secondary Endpoints

- To compare safety of the two anesthetic techniques we will document rates of moderate and severe AEs, device-related malfunctions, occurrence of device displacement, and time to complete clinical wound healing

---

[i] AEs will be defined in the study manual of operations and will be based on those found in the report of the January 2013 meeting of the WHO Technical Advisory Group on Innovations in Male Circumcision, as well as those being used in ongoing pilot implementation studies of PrePex, another male circumcision device being studied in Africa.

[ii] Complete clinical wound healing is defined as intact epithelium (unbroken skin) covering the wound as judged by the provider on visual inspection, meaning that none of the following are present: scabbing, drainage, moisture, gaps between epithelial edges or ulceration.

- To compare ease of use of the topical vs. standard injection technique for local anesthesia during Shang Ring circumcision, we will document dwell time required for onset of adequate anesthesia with the topical cream and time to adequate anesthesia with injectable anesthesia, potency of anesthesia provided by both techniques, and problems encountered during anesthesia with the two techniques.
- We will also explore service optimization in terms of determining how to best sequence and group patients for Shang Ring circumcision given the dwell time necessary for topical anesthetic effect.
- To compare duration of post-procedural analgesic effects between the topical vs. injectable anesthesia groups, we will document reported pain at various time points such as 1 and 24 hours post-circumcision between the two groups using the VAS.
- To compare satisfaction with topical vs. injectable anesthetic, we will interview study participants and their parents or legally acceptable representative (LAR) as appropriate to document acceptability and satisfaction with the circumcision, the Shang Ring procedure, post-procedure pain, and time to return to normal activity between the two techniques
- To compare satisfaction with the topical vs. injectable anesthetic among clinical providers in the study, will we will interview them about their experiences, acceptability and satisfaction with the two anesthetic techniques

4.0 STUDY DESIGN

The study, to be conducted in two phases, will examine procedural and clinical outcomes, as well as participant and provider acceptability, of adaptations of the Shang Ring technique for male circumcision that would simplify its use and increase its acceptability.

- Phase I will be non-comparative for exploration of the no-flip technique for Shang Ring circumcision (i.e. all participants will be circumcised using the no-flip Shang Ring technique). Historical data from standard Shang Ring circumcisions conducted in Africa (Kenya, Uganda and Zambia) will be used as the comparison group. Men will be randomized to removal at 7 days after circumcision vs. delayed removal, to assess occurrence and safety of spontaneous detachments following circumcision with the no-flip technique.
- Phase II will compare the use of topical vs. injectable anesthesia for Shang Ring circumcision. Participants will be randomized to topical vs. injectable anesthesia in a 2:1 ratio. We rationalize the 2:1 randomization scheme given that we will have just completed Phase I in which 200 men and boys will have been circumcised using the no-flip technique with injected anesthesia. However, given the subjectivity associated with using reported pain as the primary endpoint, we believe it is critical to randomize participants in this phase of the study.

Because of the possibility that boys and men could have different outcomes after no-flip Shang Ring circumcision or could respond differently to use of topical anesthesia, we will recruit participants equally in two age groups in both phases; 50% of participants recruited in each phase will be between 10-15 years old and the other 50% over 15 years old. Because recruitment shall be taking place at two sites, which may have different recruitment rates, and because clients need to be randomized 1:1 and 2:1 into

the Phase 1 and Phase 2 treatment groups, respectively, appropriate stratified block randomization techniques by age will be applied to achieve the intended number of participants per treatment group.

We expect the Shang Ring to receive WHO prequalification while the study is ongoing, and once this happens, there will be an interest in moving the Shang Ring into programmatic use as quickly as possible. Knowing the safety of the no-flip approach and whether or not topical anesthesia is feasible sooner rather than later may facilitate introduction of the device. Given this, we may overlap the two phases of the study if that seems logistically feasible based on study performance. For example, if it is clear that the no-flip technique is safe and acceptable at that time that recruitment is completed in the Phase I, we may begin recruitment into Phase II while follow-up is being completed in Phase I. Alternatively, because the age groups are the same in the two phases, i.e. 10-15 years old and over 15 years old, we could start Phase II in one age group as soon as recruitment into Phase I in that age group has been completed, assuming that we have sufficient evidence regarding the safety and acceptability of the no-flip technique. For example, after 115 boys less than 15 years old have been enrolled in Phase 1, we could start enrollment for Phase II in that age group. This decision will be made in consultation with the Data Safety and Monitoring Board (see section 9.3)

Below is a diagrammatic representation of the two phases of the study, which outlines the visits and procedures to be carried out during each visit.

Phase I

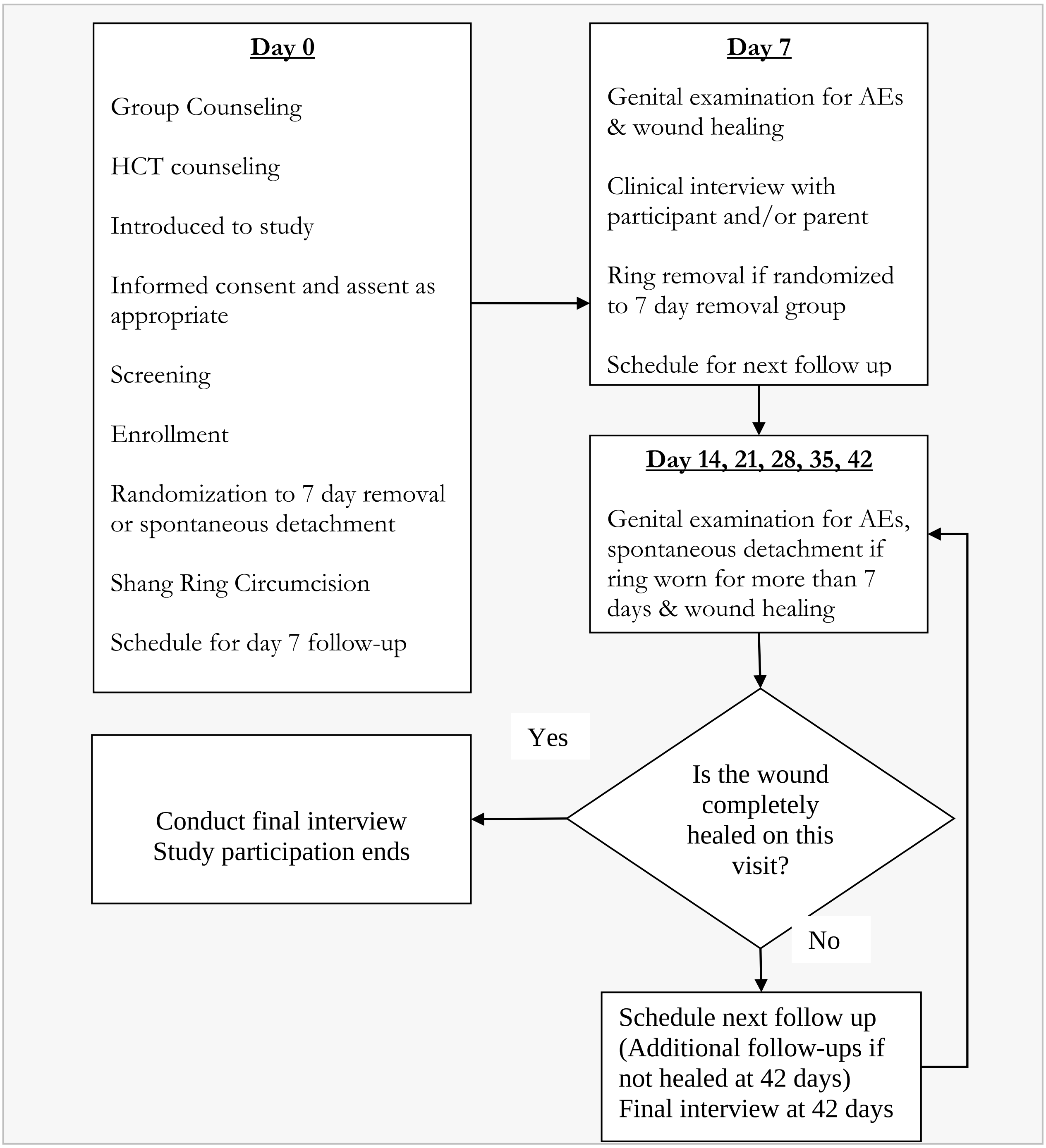
Day 0
Group Counseling
HCT counseling
Introduced to study
Informed consent and assent as appropriate
Screening
Enrollment
Randomization to 7 day removal or spontaneous detachment
Shang Ring Circumcision
Schedule for day 7 follow-up
Day 7
Genital examination for AEs & wound healing
Clinical interview with participant and/or parent
Ring removal if randomized to 7 day removal group
Schedule for next follow up
Day 14, 21, 28, 35, 42
Genital examination for AEs, spontaneous detachment if ring worn for more than 7 days & wound healing
Is the wound completely healed on this visit?
Yes
Conduct final interview
Study participation ends
No
Schedule next follow up
(Additional follow-ups if not healed at 42 days)
Final interview at 42 days

Phase II

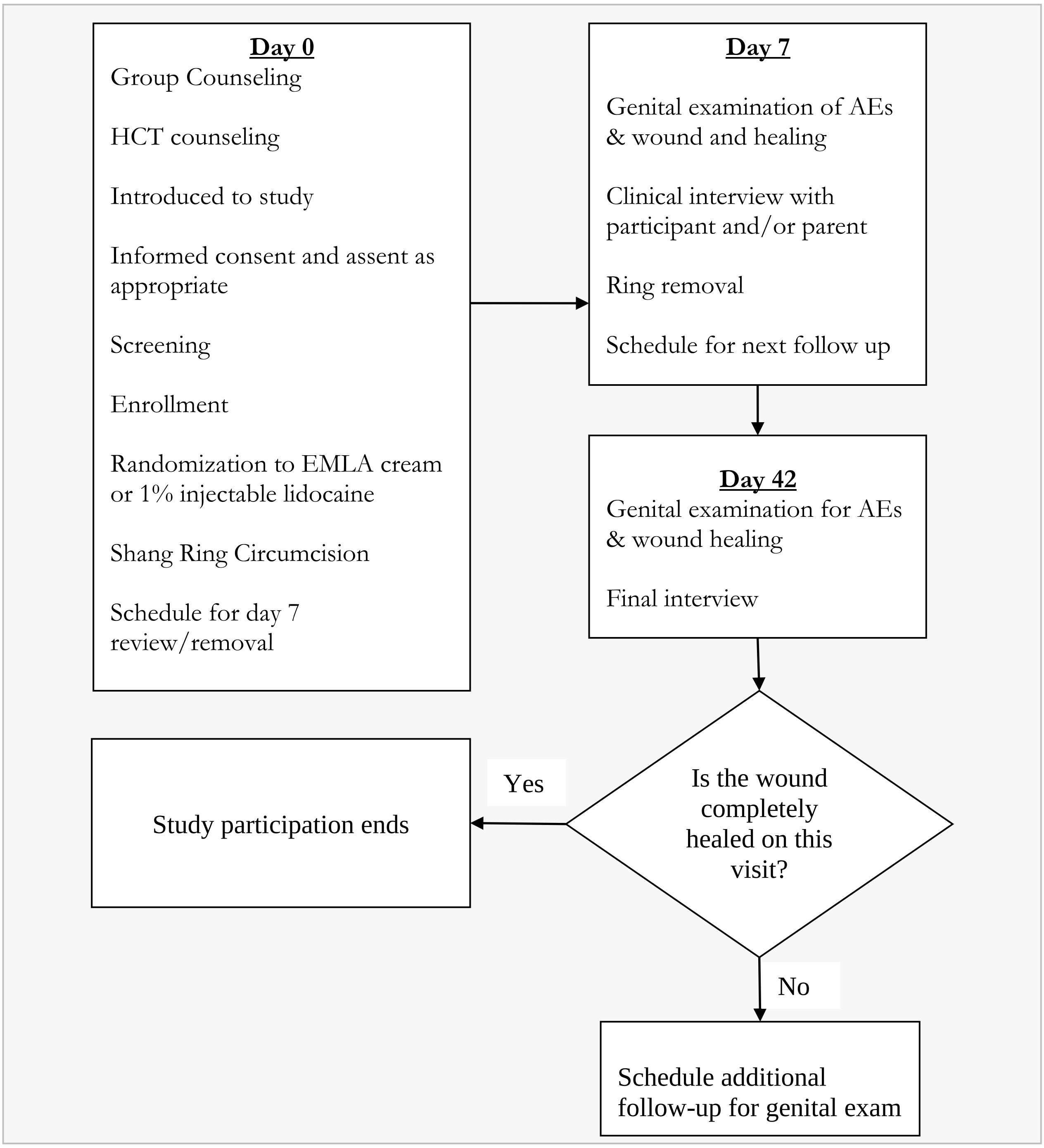
Day 0
Group Counseling
HCT counseling
Introduced to study
Informed consent and assent as appropriate
Screening
Enrollment
Randomization to EMLA cream or 1% injectable lidocaine
Shang Ring Circumcision
Schedule for day 7 review/removal
Day 7
Genital examination of AEs & wound and healing
Clinical interview with participant and/or parent
Ring removal
Schedule for next follow up
Day 42
Genital examination for AEs & wound healing
Final interview
Is the wound completely healed on this visit?
Yes
Study participation ends
No
Schedule additional follow-up for genital exam

5.0 STUDY SITE

This study will be conducted at two sites in Kenya: (1) Homa Bay County Hospital, Homa Bay County, in the western region, and (2) Vipingo Health Center, in Kilifi County, along the coast. The study sites were determined in conjunction with NASCOP VMMC National coordinator and county health authorities. Circumcisions will be done by trained medical officers, clinical officers or nurses.

Staff at the Homa Bay County Hospital have had extensive experience with use of the Shang Ring in adults, having been the site where the previous four Shang Ring studies with adults described earlier were conducted. They also have experience with Shang Ring circumcisions in boys under 18 years old as the county hospital was the site for the Shang Ring in children pilot study described above.

Staff from the site in Vipingo conduct conventional surgical circumcisions in men and boys but do not currently have experience with the Shang Ring. Clinical officers and nurses from the site will be trained on the Shang Ring procedures by staff from Homa Bay prior to study enrollment. The staff from Homa Bay have experience training other male circumcision providers on use of the Shang Ring, having previously trained providers from other facilities in Kenya, as well as from Uganda and Zambia.

6.0 STUDY PARTICIPANTS

6.1 Population Base

Participants will be recruited for the study from boys and men, 10 years and older who are seeking MC at one of the study sites. A total of 575 participants shall be enrolled into the study (230 in Phase I and 345 in Phase II). Because of the possibility that boys and men could 1) have different outcomes following no-flip Shang Ring circumcision or 2) respond differently to use of topical anesthesia, we will recruit participants in two age groups, with half of the participants in each phase being 10-15 years old and the other half being over 15 years of age.

6.2 Inclusion Criteria

All participants enrolled in this research study must meet the following inclusion criteria:

- Aged 10 years and older;
- Uncircumcised upon clinical examination;
- In good general health;
- Free of genital ulcerations or other visible signs of STI upon clinical examination;
- Participant and parent or legally acceptable representative (LAR) as applicable must be able to understand study procedures and requirements of study participation;
- Freely consents to participate in the study and signs a written informed consent form if 18 years of age or greater
- Accompanied by the parent/LAR, who freely consents and signs an informed consent form for participation of the child into the study for participants less than 18 years old;
- Assent from participant less than 18 years old who understand study procedure;

- Participant must agree to return to the study site for the full schedule of follow-up visits after his circumcision (or as appropriate the Parent or LAR must agree to bring the participant);
- Participant and parent/LAR as appropriate must agree to provide the study staff with an address, phone number, or other locator information while participating in the research study.

VCT will be offered according to the Kenya National VMMC guidelines to potential participants. Participants who undergo VCT (or the parents/LAR of those participants) may or may not make known their results to the study staff.

6.3 Exclusion Criteria

A client will be excluded from participation in this research study if he has any of the following exclusion criteria:

- Has a known allergy or sensitivity to lidocaine or other local anesthesia;
- Takes a medication that would be a contraindication for elective surgery, such as an anticoagulant or steroid;
- Has known bleeding/clotting disorder (e.g. hemophilia);
- Has any congenital genitourinary abnormality;
- Has an active genital infection, anatomic abnormality or other condition (e.g. diabetes or sickle cell anemia), which in the opinion of the surgeon, prevents the man from undergoing a circumcision as part of this study; or,
- Is currently participating in another biomedical research study.

6.4 Client Withdrawal/Discontinuation

Participants may withdraw from the study at any time without loss of services to which they may normally be entitled to at the site. Study staff members will record reasons for discontinuation. Every reasonable effort will be made to assess information relevant to study outcomes at the time of discontinuation and refer the participant for follow-up care as needed.

Once a subject has undergone a circumcision procedure, he will continue in the study for all scheduled visits, even if study recruitment is halted.

If withdrawal of a client happens reasonably before the anticipated end of the study period as a whole, recruitment efforts shall be made to replace the participant in the particular study group he belonged.

7.0 STUDY PRODUCT

7.1 The Shang Ring Device

The Shang Ring is an innovative device for MC that was invented by Mr. Jian-Zhong Shang in China, and has been on the Chinese market since 2005. The Shang Ring is manufactured at Mr. Shang's company, Wuhu SNNDA Medical Treatment Appliance Technology Co., Ltd (SNNDA). The Company license number is 340208000001506 1-1. The Chinese Medical Device registration number: Anhui Medical Device #2010, 2090013. SNNDA obtained a European Conformity (CE) mark (CE Certificate No. 08 0678 QS/NB) on October 10, 2008, which allows the Shang Ring to be sold in the

countries of the European Union. SNNDA also obtained an EN ISO certificate on November 26, 2007, which was renewed in 2012 (Canada and Norway). (Appendix IV). Notably, in September 2012, the Shang Ring received US FDA clearance (included as a separate document in the submission package).

Clinical data from Shang Ring studies in Africa were reviewed by the WHO Technical Advisory Group (TAG) on Innovations in Male Circumcision in January 2013 and were found to meet its clinical requirements (WHO 2013b). The Shang Ring manufacturer has also been accepted into the WHO Prequalification Programme for Male Circumcision Devices. The dossier of data from African studies was submitted for review and the Shang Ring manufacturing facilities were inspected by an expert team from WHO in July 2013, with a follow-up inspection in March 2014. A prequalification decision is expected in sometime in 2014. As a result of negotiations with the manufacturer, Mr. Shang has agreed to an initial public sector price of $9.00 (with potential lower pricing with large-quantity orders), which should allow for it to be used widely in national MC programs in Africa.

Over 600,000 Shang Rings have been sold in China for use in circumcising men and boys. It has been formally studied in over 3000 patients in both China and Africa (Kenya, Uganda and Zambia).

The Shang Ring is a sterile device consisting of two concentric medical grade plastic rings: an inner ring with a silicone band and an outer, hinged ring. The inner ring fits inside the outer ring which will lock when snapped together. The Shang Ring comes in multiple sizes for use with newborns down to adults. The appropriate size is determined through use of a measuring strip. As this study will be conducted on a sample with a wide range of ages (10 years and up) a wide range of sizes is anticipated to be needed based on our previous research and we will ensure an adequate supply of all rings sizes is readily available.

Below are the measurements of each of the ring sizes that would be available for use in the study.

Shang Ring size	Inner diameter of inner ring (mm)	Shang Ring size	Inner diameter of inner ring (mm)
A4	40	M	24
A3	39	N	23
A2	38	O	22
A1	37	P	21
A	36	Q	20
B	35	R	19
C	34	S	18
D	33	T	17
E	32	U	16
F	31	V	15
G	30	W	14
H	29	X	13
I	28	Y	12
J	27	Z	11
K	26	Z1	10
L	25	Z2	9

To ensure that men cannot remove the device prematurely, the locking mechanism of the outer ring can only be broken using a special lock opener. A pair of special scissors, specifically designed for this purpose, is used to cut the inner ring, hence releasing it from the shaft of the penis.

Devices will be visually inspected for breakage or other defects by the MC provider before surgery. Any defective devices will be put aside for return to the manufacturer with a description of the defect. Since the device is marketed, has a CE mark and U.S. FDA clearance and the company has ISO 13485 certification, we do not anticipate problems with defective devices. In our previous studies, we have not had any problems with defective devices.

7.2 The Shang Ring Circumcision Procedure and Removal

The standard Shang Ring circumcision technique involves a few simple steps (Appendix IV). First, a special measuring strip is used to determine which Shang Ring size to use (Step 1). Following administration of injectable 1% lidocaine for local anesthesia, the inner ring is fitted at the base of the glans penis (Step 2). In Phase II of the study, participants will be randomized to injectable 1% lidocaine or 5 grams of EMLA cream applied topically. Next, the foreskin is everted over the inner ring (Step 3) and the outer ring is secured (locked) over the inner ring (Steps 4), thus encasing the foreskin. The sterile device forms a tight seal. The foreskin is excised and several nicks are made on the incision line to prevent formation of a stiff, circumferential scab (Steps 5). There is no bleeding though a very small amount of blood may be seen from the excised foreskin. No suturing or hemostasis is required. Finally, the participant returns in seven to ten days for removal of the Shang Ring device.

In Phase I, we will be studying a variation to the procedure described above, known as the “no-flip technique” (see Appendix 1). With this variation, following measurement to determine the size and administration of local anesthesia as described above, the inner ring will be placed within the foreskin, to sit around the glans of the penis (as opposed to being fitted over the penis, at the base on the glans). Next the outer ring shall be clamped around the inner ring, on the outside of the foreskin. The foreskin shall then be excised as noted above.

The removal procedure is shown in Appendix II. Using a Shang Ring removal key, the outer ring is unhinged and removed (Step 1). The inner ring is still in place at the base of the glans penis. In Step 2, the inner ring is carefully pulled back from the edge of the wound. The inner ring is cut in two places using the special scissors designed for this purpose (Step 3). The inner ring is then removed. After removal, a bandage is applied to the wound (Step 4). Men are given a supply of bandages and told to change the bandaging daily for seven days. Men randomized to the spontaneous detachment group will not have the Shang Ring removed at the 7 day follow-up visit. Should ring removal be necessary before complete spontaneous detachment has occurred (either due to a problem/AE or because it is requested by the participant), the ring will be removed following the procedure described above.

7.3 Labeling and Packaging of Shang Ring Supplies

The Shang Ring and associated surgical supplies are packaged and sterilized using gamma radiation. Although the device is approved for use in China, has a CE Mark from the European Union, and has received U.S. FDA clearance, it is not yet approved for use

for medical male circumcisions in Kenya. It is, therefore, classified as an investigational device and will be labeled[iii] as follows:

<table>
<tr><td colspan="2" align="center">RESEARCH DEVICE: SHANG RING

CAUTION: INVESTIGATIONAL DEVICE. LIMITED TO INVESTIGATIONAL USE
This product requires training for correct use. The Shang Ring device should not be used by medical personnel in the provision of male circumcision unless product specific training has been provided in the procedure by an experienced healthcare professional. The Shang Ring device must not be used outside of the research-study setting.</td></tr>
<tr><td>DEVICE DISTRIBUTED BY:
EngenderHealth
P.O. Box 57964
ABC Place, 1st floor,
Waiyaki Way
Nairobi, Kenya</td><td>Quantity: 1
Lot #:
Expiration Date:</td></tr>
</table>

7.4 Device Accountability

7.4.1 Device Shipment and Handling

EngenderHealth will facilitate the shipment of the Shang Ring from China to Kenya. The device manufacturer has already agreed to provide the devices and associated supplies (measuring tapes, bandages, and removal key and scissors) for the study at no cost other than the shipping fees.

Documentation of shipment, including shipping conditions, and receipt in Kenya will be kept at the study site.

All supplies necessary for standard, surgical circumcision methods are already present in country and available at the study site. Thus, no additional imports, beyond the Shang Rings and associated supplies, are needed.

7.4.2 Product Tracking

In Kenya, study products will be labeled, counted, and inventoried by EngenderHealth staff and verified by a monitor at every site monitoring visit.

Inventory information about the use of the rings and the client (numbers) to whom they have been dispensed to will be recorded in a ring inventory log. This log will be stored in the study regulatory binder at the end of the active study recruitment.

7.4.3 Product Tracking and Disposition after Use

At the time of device removal or spontaneous detachment, we will note on the electronic CRF the disposition of the device. The Shang Ring removal technique also involves destruction of the device. Specifically, the outer ring locking mechanism is broken and the inner ring is cut into two pieces, rendering the device unusable. For cases where the device detaches spontaneously, the client or the client's parent/LAR will be asked to bring the device to the clinic, and it will be noted on the electronic CRF whether or not they returned the devices in such cases. In the case of spontaneous detachment, devices that are returned to the clinic by participants will be broken as

[iii] Please note that the actual label will be much smaller to accommodate the size of the packaging.

described above, to prevent re-use. After removal/destruction, devices will be disposed of using the routine procedure for other medical waste at the study sites.

7.5 EMLA Cream

EMLA is an acronym representing Eutectic Mixture of Local Anesthestics. Eutectic mixtures permit higher concentrations of anesthetic to be used safely and facilitate application to the skin. EMLA is a 5% mixture of equal quantities by weight of lidocaine and prilocaine. When used separately, lidocaine and prilocaine are solid bases but when mixed in equal quantities, however, they form a mixture that results in an oil that allows for higher concentrations of anesthetic to be maintained during application and permits better absorption leading to greater potency but with reduced toxicity. This may enhance the anesthetic effect of EMLA over other topical anesthetic preparations. EMLA cream has been approved by the U.S. Food and Drug Administration since the early 1990s and is commercially available at reasonable cost in Kenya, as well as other countries in sub-Saharan Africa that have prioritized MC as an HIV prevention intervention, making it a good option for use with the Shang Ring if shown to be safe an effective.

The use of topical lidocaine, including EMLA, has historical precedent in device-driven circumcision, as reported for the PrePex and Unicirc devices (Mutabazi et al., 2013, Bitega et al., 2011. See video at https://www.youtube.com/watch?v=e3i1MIhV1ZU). One should note that the topical anesthetic used in the PrePex publications was not EMLA, but rather a "homemade" lidocaine cream. As studies progressed, the impractical nature of using such a cream for scale-up was recognized, and the ongoing PrePex pilot implementation studies in several sub-Saharan Africa countries are now using EMLA. The use of EMLA cream has also been extensively reported to be safe and effective in neonatal circumcision (Taddio, 2001 and American Academy of Pediatrics Task Force on Circumcision, 2012)

We will apply EMLA prior to circumcision with the Shang Ring, allowing for a dwell time of up to 45 minutes for onset of action, under an occlusive dressing. As noted below under section 8.2 we will explore time course of anesthetic effectiveness with a small number of men at the start of Phase II to get a better sense of minimum required dwell time.

8.0 STUDY VISITS & PROCEDURES

> Procedures will be generally similar at each visit in the two phases of the study. Specific differences are noted in the sections that follow.

8.1 Recruitment

Participants will be recruited for the study from boys and men, 10 years and older who are seeking MC at one of the study sites. A total of 580 participants shall be enrolled into the study (230 in Phase I and 345 in Phase II). Because of the possibility that boys and men could have different outcomes following no-flip Shang Ring circumcision or could respond differently to use of topical anesthesia we will recruit participants in two age groups, with half of the participants in each phase being 10-15 years old and the other half being over 15.

VCT shall be offered to the clients according to Kenya's national guidelines.

Clients, with their accompanying parents/LAR when less than 18 years old, will be introduced to the study. They will be informed that the study involves Shang Ring circumcision and be familiarised with the Shang Ring.

8.2 Screening, Enrollment, and Circumcision Visit

During the screening visit, study staff will explain the study in detail to prospective participants and their parents/LAR, as appropriate. Each participant and parent/LAR as appropriate will be asked to sign the informed consent form to document his voluntary decision to participate or his/her voluntary decision to have his/her child in the study. Assent from the potential study participant will be obtained as appropriate (see detailed discussion on informed consent under section 12.2). After the informed consent form (and assent form, as appropriate) has been signed, each potential participant will be evaluated for clinical eligibility according to the study inclusion and exclusion criteria. If eligible, the enrollment procedures will be done on the same visit.

The following will be done at the screening/enrollment visit:

- Administer informed consent and as appropriate assent;
- Assign the client a participant number;
- Confirm eligibility
- Administer study questionnaires and data forms to collect medical, surgical and relevant sexual histories and demographic information about the participant
- Conduct a directed physical examination, including a genital exam;
- Review concomitant medications; and,
- Collect contact information from the participant and/or his parent/LAR.

In most cases, the circumcision procedure will be done on the day of enrollment. If for whatever reason a participant cannot be circumcised on the day of consent, physical examination and qualification, the participant will be asked to return (or the parent/LAR asked to return with the participant) to the clinic within one week (seven days) to complete the circumcision procedure. Participants <u>not</u> returning within one week of screening will not be admitted into the study without re-screening.

The following is to be done on the day of circumcision (within 7 days of screening and enrollment):

- Shang Ring circumcision procedure;
- Assessment of pain during and post-circumcision procedure.
- Review wound care and post-circumcision instruction with the parents/LAR and participant as appropriate.

Phase I

In Phase I, the Shang Ring circumcision will be completed using the no-flip technique described above for all enrolled participants. Participants will be randomized 1:1 prior to the start of the circumcision procedure to removal at 7 days or spontaneous detachment. Local anesthesia will be induced via dorsal penile nerve block (DPNB) using 1% lidocaine without epinephrine, as per WHO recommendations (WHO 2008) and Kenya national VMMC practice. This is what has been used for local anesthesia in our previous Shang Ring studies in Africa. Because of the possibility

that boys and men could have different outcomes, we will recruit participants equally in two age groups; 50% ages 10-15 years old and 50% over 15 years old.

Phase II

Assuming positive outcomes of Phase I, we will use the no-flip technique for all Shang Ring circumcisions in Phase II.[iv] In this phase, participants will be randomized to topical or injectable anesthesia in a 2:1 ratio. We rationalize the 2:1 randomization scheme given that we will have just completed Phase I in which 230 men and boys will have been circumcised using the no-flip technique with injected anesthesia. However, given the subjectivity associated with using reported pain as the primary endpoint, we believe it is critical to randomize participants in this phase of the study. Because of the possibility that boys and men could respond differently to use of topical anesthesia we will recruit participants equally in two age groups; 50% ages 10-15 years old and 50% those over 15 years old.

To get a better sense of minimum dwell time necessary for adequate anesthesia, we will test for anesthetic effect more frequently (e.g. every 3-5 minutes) in the first 10 participants randomized to the topical arm in each age group (10-15 and over 15 years old), as well as any participants randomized to the injectable arm during this period. During this time we will also closely monitor the occurrence of any significant problems with application of the topical anesthesia or potency of the anesthesia with the topical agent. This will allow us to establish a minimum dwell time which will be important for logistic and program planning purposes, although each participant will be tested for numbness as described below before the circumcision procedure is started. Given the previous experience with use of EMLA cream for male circumcision, we are expecting that it will provide adequate anesthesia for Shang Ring circumcision. Should we determine that in fact it does not, or that there are unexpected problems, we may explore other options such as 4% or 5% lidocaine cream.

The injectable approach shall utilize a combination of a penile and a ring block with 1% lidocaine injected with a small gauge needle. This is the standard anesthesia approach used in the Kenya VMMC program. The maximum amount of lidocaine administered will be 4.5 mg/kg. Onset of action typically requires 3 to 10 minutes. The topical approach shall consist of applying EMLA cream to the both the inside and outside surfaces of the foreskin. The anesthetic will then be allowed to dwell for up to 45 minutes for onset of action, with appropriate adjustments made based on the run-in described in the previous paragraph.

In both the injectable and topical anesthetic groups, we will test for numbness using the approach that is currently used, i.e. using blunt forceps on all of the quadrants of the penile shaft, on both the cutaneous and the mucosal sides to determine if anesthesia has taken effect. If it is determined that anesthesia has not taken effect in any participant in the topical group, we will resort to injectable anesthesia.

[iv] Given the experience with the no-flip technique in China and our experience with the technique in the pilot study on use of the Shang Ring in children in Kenya, we are not expecting any outcomes that might suggest that the no-flip technique is inappropriate, however, should that be the case, we will use the standard Shang Ring technique for circumcisions in Phase II.

The Wong-Baker FACES Visual Analogue Scale (VAS) shall be used for pain assessment as we have done in our previous Shang Ring studies (Barone et al., 2011; Barone et al., 2012; Barone et al., 2013, Sokal et al., 2014).

The immediate complications of the circumcision procedure will be recorded on either an Adverse Event (AE) or Serious Adverse Event (SAE) form and may include such findings as: excessive pain, excessive bleeding, anesthetic-related complications, excessive swelling, excessive skin removal, and damage to the penis (e.g. injury to the glans or urethra). Data on procedure times and surgical difficulties, including problems with use of Shang Ring shall also be collected.

Participants will be instructed to return to the study site if they experience medical events such as severe discoloration of the penis, signs of infection (purulence), severe or increasing pain, or any other significant concern that they may have.

Study staff will contact participants or their parents/LAR if appropriate by mobile phone call and/or SMS to remind them of their next follow-up visit. All reasonable efforts will be made to contact those who do not return for follow-up visits. If necessary home visits may be resorted to especially in the case where the ring has not yet been confirmed at the site to have been removed or the client has an AE.

8.3 Post-Op Follow-Up Visits

Scheduled follow up shall be conducted for a maximum of 42 days in both phases of the study, although the follow-up schedule will differ as noted below. If needed, weekly visits after Day 42 will continue until complete healing is documented in both phases.

Phase I: Participants will be asked to return to the study site for follow up on days 7, 14, 21, 28, 35, and 42. However if a client is healed on an earlier visit, e.g. day 21, his participation in the study will be completed at that time. There will be a clinical exam at each follow-up visit and an interview with the participant and/or his parent/LAR.

Phase II: Participants will be asked to return for two follow-up visits on days 7 and 42 post-circumcision. Given that we will have just completed 200 no-flip Shang Ring circumcisions with intensive follow-up as well as the data from our other previous Shang Ring vs. conventional MC RCTs using the standard Shang Ring technique, we will ask participants to return for only one follow-up visit after ring removal, at 42 days following the circumcision procedure. We have no reason to believe that the type of anesthesia used for the circumcision procedure should have any impact on the course of healing or other outcomes after removal of the device on day 7.

The informed consent form will explain to participants that we may take photographs of their penis at various points during the preparation for and completion of the Shang Ring circumcision or device removal, or at one or more follow-up visits. However we will always ask the participants and parent/LAR as appropriate for permission immediately before taking any photographs, and participants will not be discontinued from the study if permission is not granted to proceed with photography. We intend to take photographs of the circumcision and removal procedures, of participants that may incur an AE and at follow-up visits to document wound healing.

At follow-up visits, clinical outcome measures will include adverse events and early post-surgical complications. These include the following:

- pain,
- excessive bleeding,
- problems with the delayed healing (understood herein as being beyond 42 days),
- swelling or hematoma,
- damage to the penis (e.g. injury to the glans or urethra),
- infection,
- problems with the appearance of the penis/poor cosmetic results,
- torsion,
- erectile dysfunction,
- problems with voiding,
- any other complaint from the client, including social harm events.

Participant (or parent/LAR as appropriate) interviews will cover a range of topics, including acceptability of the device and pain of the procedure and outcome, satisfaction with procedure, problems encountered both before and after device removal, compliance with post-surgical instructions.

During the scheduled follow-up visits post circumcision, the following tasks, activities or documents will be completed:

**Phase I**	
**Day of Visit**	**Study procedures to be conducted**
Day 7	1. Remove Shang Ring if randomized to 7 day removal group<br>2. Conduct genital examination;<br>3. Examine penis and assess wound healing;<br>4. Complete relevant eCRFs;<br>5. Review AEs and SAEs, if applicable;<br>6. Review concomitant medications, if applicable;<br>7. Take penile photographs if participant agrees;<br>8. Schedule the next follow-up visit;<br>9. Reimburse participant/parent/LAR for transport costs.
Day 14, 21, 28, 35 and 42	1. Conduct genital examination;<br>2. Assess for spontaneous detachment if randomized to spontaneous detachment group<br>3. Examine penis and assess wound healing;<br>4. Complete relevant eCRFs for the visit;<br>5. Review AEs and SAEs, if applicable;<br>6. Review concomitant medications, if applicable;<br>7. Take penile photographs if participant agrees;<br>8. Schedule the next follow-up visit (participants will be asked to return for additional follow-up if they are not completely healed by the 42 day visit);<br>9. Administer final study interview if client has healed or at day 42<br>10. Reimburse participant/parent/LAR for transport cost.

Phase II	
**Day of Visit**	**Study procedures to be conducted**
Day 7	1. Remove Shang Ring<br>2. Conduct genital examination;<br>3. Examine penis and assess wound healing;<br>4. Complete relevant eCRFs;<br>5. Review AEs and SAEs, if applicable;<br>6. Review concomitant medications, if applicable;<br>7. Take penile photographs if participant agrees;<br>8. Schedule the next follow-up visit;<br>9. Reimburse participant/parent/LAR for transport costs.
Day 42	1. Conduct genital examination;<br>2. Examine penis and assess wound healing;<br>3. Complete relevant eCRFs;<br>4. Review AEs and SAEs, if applicable;<br>5. Review concomitant medications, if applicable;<br>6. Take penile photographs if participant agrees;<br>7. Administer final study interview<br>8. Reimburse participant/parent/LAR for transport cost.

8.4 Interim and Unscheduled Visits

Additional visits may occur in either phase of the study as needed for complications, participant/parent/LAR concerns or to assess wound healing that is delayed beyond 42 days. Participants and their parents/LAR as appropriate will be told they should come for an unscheduled visit if they experience medical events such as: difficulty urinating, increasing swelling with discoloration of the penis, signs of infection (purulence), severe or increasing pain, or other significant concern. Participants and their parents/LAR as appropriate will also be asked to return to the clinic if they experience any untoward symptoms from the ring or any problems while wearing the ring that causes them significant concern. At interim or unscheduled visits, the following tasks, activities or documents will be completed:

Day of Visit	Study procedures to be conducted
Interim/ Unscheduled visit	1. Conduct genital examination<br>2. Examine penis and assess wound healing;<br>3. Review AEs and SAEs, if applicable;<br>4. Review concomitant medications, if applicable;<br>5. Take penile photographs if participant agrees;<br>6. Schedule additional follow-up visits if necessary.

8.5 Summary of Activities at each Study Visit

Phase I

STUDY ACTIVITY	Day 0 Visit	Day 7 visit	Day 14, 21, 28, 35, 42 Visits	Final Visit
Informed Consent Process	X			
Assign Participant Number	X			
Physical Exam and Medical History	X			
Genital Exam	X	X	X	X
Screening Checklist	X			
Demographic Survey	X			
Obtain Locator Information	X			
Circumcision Procedure	X			
Shang Ring Device Removal (for those randomized to the 7 day removal group)		X		
Assess Pain and/or AEs or SAEs	X	X	X	X
If permitted take Penile Photograph	X	X	X	X
Review Concomitant Medications	X	X	X	X
Schedule follow-up if wound not healed	X	X	X	
Reimburse Participant		X	X	X
Final Status Form				X

Phase II

STUDY ACTIVITY	Day 0 Visit	Day 7 Visit	Day 42 Visit
Informed Consent Process	X		
Assign Participant Number	X		
Physical Exam and Medical History	X		
Genital Exam	X	X	X
Screening Checklist	X		
Demographic Survey	X		
Obtain Locator Information	X		
Randomization	X		
Circumcision Procedure	X		
Shang Ring Device Removal		X	
Assess Pain and/or AEs or SAEs	X	X	X
If permitted take Penile Photograph	X	X	X
Review Concomitant Medications	X	X	X
Schedule Follow-Up Visit	X	X	
Schedule follow-up if wound not healed			X
Reimburse Participant		X	X
Final Status Form			X

8.6 Participant Compensation

Participants or their parents/LAR will be given a small cash stipend to cover transport cost to and from the study site for the scheduled follow-up visits. This will not include the first (day 0) circumcision day visit. The amount of the stipend will be 350 Kenyan Shillings (KES), the same amount we have used in our previous Shang Ring studies in Homa Bay. Stipends will not be disbursed for unscheduled/interim visits other than those scheduled due to delayed healing beyond 42 days. As is the case with circumcisions provided as part of the national VMMC program, the circumcision procedure and follow-up care will be provided at no cost.

9.0 STATISTICAL CONSIDERATIONS

9.1 Study Size Justification

By recruiting 230 participants into Phase I of the study we expect to have complete follow-up data on at least 200. This sample size number is in line with the recommendations from the World Health Organization for clinical evaluation of MC devices, which is based on an ability to rule out an AE rate of about 5% with a new use of a device that has already been shown to be safe in a larger group of men in RCTs and demonstration studies. (WHO 2012)

In Phase II, by recruiting 345 participants, we expect to have complete follow-up data on at least 300. For comparing postoperative pain scores, we will randomize 345 males in 2:1 ratio to topical vs. injectable anesthesia for circumcision. This sample would provide 100% power to detect a mean difference of 1 on the 0 to 10 pain scale using a 2-sided test at the 0.05 significance level. This calculation assumes a standard deviation of 1.8, which is based on prior data. For other binary endpoints, this sample size will provide at least 80% power to detect absolute differences in proportions between groups of at least 20% separately within each site using two-sided tests at the 0.05 significance level. We will not make any adjustments for multiple comparisons between randomized groups.

9.2 Analysis Plan Summary

A detailed analysis plan that covers both the final analysis and the planned interim analyses will be developed and reviewed and approved internally prior to initiation of the analysis. The following is a summary of the planned analyses for the two phases. Any deviations to be made from this summary plan will be documented in the detailed analysis plan.

In Phase I, the frequency and percentage of men with procedure-related AEs will be tabulated by severity, both overall and by site. Listings of AEs will include information on duration, outcome, severity, and relation to study procedures. Listings of any serious adverse events will also be provided. Shang Ring circumcision and removal procedure data (e.g. time of circumcision and removal, problems encountered, ring sizes used) will be descriptively summarized. Data on operative and post-operative pain, time to return to normal activity, participants' opinions of the Shang Ring procedure and participants' satisfaction with the post-circumcision cosmetic results will be descriptively summarized. Time to complete wound healing will be evaluated using a Kaplan–Meier estimator.

As Phase II is a randomized study of acceptability and safety, primary analyses will be conducted using an as-treated approach (i.e., depending on anesthesia method actually received). However, any deviations between the as-treated approach and a strict intention-to-treat approach will be presented to allow for the assessment of potential biases. Some secondary analyses on safety endpoints may be repeated using an intention-to-treat approach, as relevant.

VAS pain scores will be compared between randomization groups to estimate the efficacy of anesthetic technique at the time of surgery. The frequency and percentage of men with AEs will be tabulated by randomization group, relation to study procedures, and by severity, both pooled across sites and separately within each site. The analysis of AEs will include information on duration, outcome, and relation to study procedures. Stratification by severity will also be considered.

All other endpoints will be summarized by anesthetic technique and compared between groups using either two-sample t-tests or chi-squared tests, as appropriate for the endpoint, all conducted at the two-sided 0.05 significance level. We will not make any adjustments for multiple comparisons. 95% confidence intervals for differences between groups will be presented. Missing data will be assumed to be missing in random fashion and ignored.

9.3 Interim Analysis

We will recruit a three member Data Safety and Monitoring Board (DSMB), including a surgeon, an epidemiologist, and a statistician, to review interim analyses for both phases of the study. They will meet before the study begins and then for each of the two interim analyses described below. Meetings will be by teleconference or in-person, as agreed to by the group. Other meetings could be schedule at the discretion of the DSMB should a problem arise at any point during the conduct of the study. The DSMB will follow guidelines and procedures proposed as part of the DAMOCLES Charter (DAMOCLES Study Group 2005).

This study will include one interim analysis for each phase.

- For Phase I, the interim analysis will be conducted after 40 men have been circumcised using the no-flip technique and had their Shang Rings removed at the 7 day follow-up visit. At the time of the interim analyses, safety, study quality, and possibly some acceptability data will be provided to the DSMB.

  The following stopping guidelines are proposed for the DSMB regarding adverse events in the first phase of the study:

  1. At the time of the interim analysis, the DSMB may recommend stopping the study or temporarily halting recruitment if there are notably higher rates of severe adverse events than have been seen in previous Shang Ring studies conducted in Africa that will serve as historical control Shang Ring cases (the DSMB will decide the level of significance they will use). In this case, the DSMB may direct that the second phase of the study be conducted using the standard Shang Ring technique.

  2. In the event that the interim analysis shows notably fewer moderate and severe adverse events among no-flip Shang Ring cases compared to the standard Shang

Ring technique, the study should continue in order to gather additional data on the no-flip Shang Ring clinical profile, such as time to healing and potential rare adverse events.

- For Phase II, the interim analysis will be conducted after 40 men have been circumcised in each age group in the topical arm (10-15 and over 15 year olds). At the time of the interim analyses, data on reported pain experienced during the circumcision, safety, study quality, and possibly acceptability will be provided to the DSMB by unmasked treatment group. Although this phase is necessarily open-label, the Lead Study Biostatistician will remain blinded to treatment group until the final analyses have been verified. An independent biostatistician, not otherwise involved with Phase II of the study, will prepare the unmasked tables for the DSMB.

  The following stopping guidelines are proposed for the DSMB regarding reported pain experienced during the procedure and adverse events in the second phase of the study:

  1. At the time of the interim analysis, the DSMB may recommend stopping the study or temporarily halting recruitment if there are notably more severe adverse events among cases using topical anesthesia compared to those using injectable anesthesia (the DSMB will decide the level of significance they will use).

  2. In the event that the interim analysis shows notably fewer moderate and severe adverse events among topical anesthesia cases compared to participants receiving standard injectable anesthesia, the study should continue in order to gather additional data on outcomes following use of topical anesthesia with the Shang Ring, such as time to healing and potential rare adverse events.

If the study is stopped temporarily or permanently for any reason, follow-up of participants already enrolled will continue for all the planned follow-up visits; longer if necessary to document complete healing. Enrolled participants will receive continued clinical care as necessary.

It should be noted that if there appears to be an unexpectedly high number of serious adverse events in either phase of the study, any one of the study IRBs may temporarily or permanently halt the study at any time. In addition to IRB oversight, the study clinicians, site PIs and the PEC will review all SAE's as well as severe, related adverse events and closely track other AEs throughout the study.

10.0 MANAGEMENT OF INTERCURRENT EVENTS

10.1 Loss to Follow-Up

A participant will not be classified as lost to follow-up until the study site is closed out.

The need to attend all scheduled study visits will be emphasized during recruitment and enrollment and at each follow-up visit. **Upon failure to show up for a scheduled study visit, attempts to contact the participant or his parent/LAR will be made within 24 hours of the missed appointment**. Attempts to follow-up will continue and may include using the alternate contacts provided during recruitment.

All missed appointments would be rescheduled to a time that is convenient for the participant and consistent with site duty hours.

All attempts to contact the participant will be recorded in the participant's file. Attempts to contact the participant through his parent/LAR will be handled discretely to ensure confidentiality and privacy.

10.2 Adverse Events

A study clinician will evaluate each adverse event for severity, seriousness and relationship to the study procedure under investigation. For the purpose of this study, the male circumcision procedure is considered to be the study procedure by which the relatedness of an adverse event will be evaluated. Because the intervention under study is a surgical procedure, it will usually be obvious whether an adverse event is related or unrelated to the procedure.

How to assess, in a standard way, the severity and seriousness, classification of relationship to procedure will be outlined in the Study Manual. Study clinicians at both sites will be trained to make these assessments in the same way in order to minimize variation.

Adverse events occurring during this study will be recorded on an Adverse Event Form. This form will include a description of the onset, duration, relationship to the procedure, severity, seriousness, and final outcome of the AE. However, common post-operative findings that are (1) clearly related to the circumcision procedure and (2) are considered within normal limits or "mild" based on the standard descriptions provided in the study manual, will be reported on follow-up visit forms rather than separate AE forms.

Classification of adverse events and device hazards related to the circumcision procedure will be based on the definitions outlined by the WHO Technical Advisory Group on Innovations in Male Circumcision (WHO, 2013b). A detailed description of these possible AEs will be fully outlined in the Study Manual.

For other adverse events not described in the Study Manual, each event reported will be graded on a three-point scale of severity:

- Mild: awareness of event, but easily tolerated
- Moderate: discomfort enough to cause interference with usual activity
- Severe: inability to carry out usual activity.

10.3 Serious Adverse Events (SAE)

Serious Adverse Events are defined as untoward medical occurrence that results in:

- Death
- Is life threatening
- Requires inpatient hospitalization or prolongation of existing hospitalization
- Results in persistent significant disability/incapacity
- Requires intervention to prevent permanent impairment or damage
- May jeopardize the patient and require medical or surgical intervention to prevent one of the above listed outcomes.

A planned hospitalization for a pre-existing condition is not considered to be a serious adverse event.

The PI or his designee must report all serious adverse events to the protocol executive committee (PEC) *within 24 hours* of becoming aware of an event that classifies an AE or SAE.

The PI or designee should complete an SAE Report Form and email it to the following:

Protocol Executive Committee	Contacts
**Quentin D. Awori, MBChB**<br>EngenderHealth Nairobi, Kenya	QAwori@engenderhealth.org
**Raymond Otieno, MBChB, MMED (Surg)**<br>MoH, Homa Bay, Kenya	drrraysimba@gmail.com
**Mark A. Barone, DVM, MS**<br>EngenderHealth, NY, USA	MBarone@engenderhealth.org
**Philip S. Li, MD**<br>Weill Cornell Medical College, NY, USA	psli@med.cornell.edu
**Richard Lee, MD**<br>Weil Cornell Medical College, NY, USA	ril9010@med.cornell.edu
**Marc Goldstein, MD**<br>Weil Cornell Medical College, NY, USA	mgoldst@med.cornell.edu

All SAEs resulting in hospitalization or death will be reported to the IRBs **within <u>10 days</u> of notification to the Protocol Executive Committee of the event for hospitalization and within <u>24 hours</u> of any research participant's death, unless otherwise required by the IRBs.**

The schematic below outlines the reporting flowchart of SAEs.

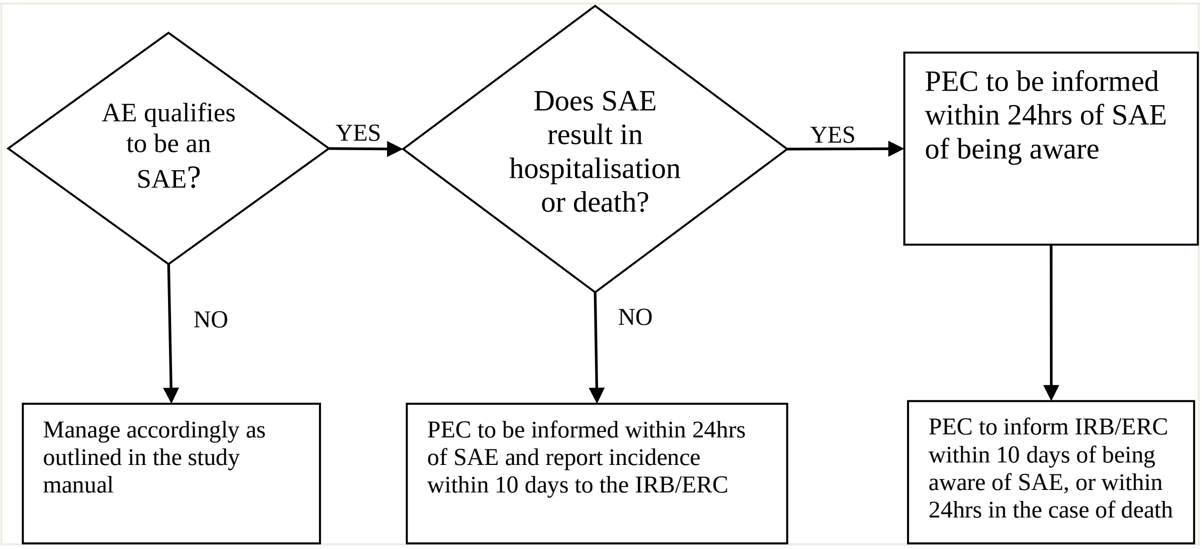

10.4 Social Harm Events

Social harm events (SHEs) may include loss of privacy, stigmatization, relationship difficulties, physical or verbal abuse, interference with gainful employment and bullying in school. Any similar experiences by the parent/LAR as a result of their child's participation in this study shall also be considered as a SHE.

Social harms may be identified during interviews with the participants or by other study staff. All SHEs (i.e., events potentially related to study participation) will be brought to the attention of the site investigator. Participants who report social harms will be referred to speak with a study staff person.

The site investigator or his/her designee will report the event on the Social Harm Event form (attachments) **to the PEC within 24 hours** of becoming aware of the same (similar to SAEs) and the PEC is to report this to the IRB/ERC within 10 days of being aware of the SHE.

It is not anticipated that there will be social harms to participants in this research study. We have not seen any such instances in our prior male circumcision or Shang Ring studies.

11.0 PROTOCOL NONCOMPLIANCE

Protocol noncompliance (whether considered as protocol deviations or violations) may be identified by the study staff or the clinical monitor during the periodic and closeout monitoring visits as well as during in-house monitoring. The procedures for capturing, recording and reporting protocol violations will be specified in the study manual and data management plan.

In general, more serious departures from the protocol will be considered violations; protocol violations affect the participant's rights, safety or welfare, or the integrity of the resultant data. Deviations are more general cases of less serious noncompliance with the protocol.

Emergency departures from protocol that eliminate an apparent immediate hazard to participants and are deemed crucial for the safety and well-being of that participant may be instituted for that participant only by the Site Principal Investigator or his/her designee. In those cases, the Site Principal Investigator will notify the PEC within 24hrs, which will notify the ERC/IRB in writing within **ten working days** after the emergency occurred. Due documentation in the Protocol Violation Form (attachments) of the reasons for the violation and ensuing events will be done.

Non-emergency deviations from the protocol must be approved by the PEC in writing prior to their implementation. If these changes might affect the scientific soundness of the protocol or the rights, safety, or welfare of human subjects, ERC/IRB approval is also required prior to implementation [21 CFR 812.150(a) (4)].

Deviations from the protocol by participants will not be considered protocol violations (e.g. not following wound care instructions; missing a follow-up visit, etc.). Study staff

will provide continued counseling of and education for the participants who do not adhere to the prescribed study procedures. This protocol may not be modified without notification and written approval of the PEC. Further, any modification must be approved by the ERC/IRBs. Exception will be made in cases where it is necessary to eliminate immediate hazards to human participants.

12.0 PROTECTION OF HUMAN SUBJECTS

12.1 Institutional Review

This research study will not be implemented without documented approval by the Kenya Medical Research Institute Ethical Research Committee (KEMRI ERC) and the Weill Cornell Medical College Institutional Review Board (WCMC IRB). The Principal Investigator (PI) will need written documentation, signed by the IRB/ERC Chairperson(s), stating that the committee has approved the research protocol and the informed consent form. The PI will also need the membership roster of both review committees, including each member's affiliation and qualification. The name of the Chairperson should be clearly noted. In addition, regulatory approval will be sought from the Kenya Poisons and Pharmacy Board prior to the start of the study.

The PI will be responsible for making safety and progress reports to the KEMRI ERC and the WCMC IRB on an annual basis and/or within three months after study termination or completion. These reports should include the total number of participants recruited and completing the study, all changes in research activity, as well as all unanticipated problems involving risk to human participants. All correspondence with the local IRB regarding this research study must be retained in the site's regulatory binder.

12.2 Informed Consent and Assent

A participant will not be admitted into this research study until the site PI or his/her designee has obtained the legally effective (signed and/or witnessed) informed consent or that of parent or LAR (e.g. guardian or others with parental responsibility) in the case of minors. In the discussion of Informed Consent, 'minors' "are persons who have not attained the legal age for consent to treatments or procedures involved in the research, under the applicable law of the jurisdiction in which the research will be conducted." The legal age of consent in Kenya is 18 years and thus some participants in this study (those between 10-17 years old) will be under the legal age of consent.

The site PI or his/her designee shall seek such consent only under circumstances that provide the prospective participant and his parent/LAR (for participants under 18 years of age) with sufficient opportunity to consider whether or not to participate in the research study. Participants and parents/LARs will be informed of all the features of the research that may affect their willingness participant or to allow their child to participate. Informed consent will be obtained without coercion, undue influence or misrepresentation of the potential benefits and risks that might be associated with participation in the research study.

For boys who are able to understand the study procedure, assent shall be required; parental/LAR consent will not substitute for the minor's own consent, but will supplement it. As we have done in our previous Shang Ring study in boys, assent shall be sought in boys between 13 and 17 years old. Boys between 10 and 13 years old who

are able to comprehend study procedure may also assent before participation in the study. Before seeking assent from any minor, the site PI or his/her designee, will inform the boy of all features of the research that may affect his willingness to participate and will answer his questions in terms appropriate to the child's comprehension. The study staff will respect the boy's freedom to choose to participate in the research or not by giving him the opportunity to give or not give assent to participation, as well as to choose to discontinue participation at any time. Assent means that the child shows some form of agreement to participate without necessarily comprehending the full significance of the research necessary to give informed consent. In the event that an understanding child refuses to take part in the study, and hence does not give assent, he will not be enrolled into the study.

Informed consent encompasses all written and oral information that will be given. This includes the consent form itself, information on the circumcision study products and procedure, and if applicable, advertising or educational materials. All such information will be in a language that is understandable to the prospective participant and, as appropriate, his parent/LAR. The Informed Consent Form will be translated into Kiswahili, which is the national language in Kenya. It will also be translated into Dholuo & Giriama, the local languages spoken by the vast majority of potential study participants in and around where the study sites will be located. The information will not include any language in which the participant or as appropriate his parent/LAR is made to waive any of his/her rights or which releases, or appears to release the investigators' institutions from liability for negligence.

Informed consent will be documented through use of a written consent form that is signed by the participants and, as appropriate, his parent/LAR, as well as the site PI or his/her designee. When appropriate, the boy will also sign an assent form. A copy of the signed consent form (and assent form as appropriate) will be given to the participants and parent/LGA. The original signed consent form(s) for each participant will be kept in the site's study files. The informed consent form includes each of the basic and additional elements of informed consent described in 21 CFR Part 50.25. The informed consent describes each of the risks and discomforts that have been identified as reasonably foreseeable.

12.3 Risks and Benefits

12.3.1 Risks of the Shang Ring Male Circumcision Procedure

Potential complications of the Shang Ring circumcision include reaction to the anesthetic agent (injectable or topical), pain, excessive bleeding, hematoma formation, infection, injury to the glans or urethra, excessive or insufficient skin removal, and poor cosmetic results. However, when Shang Ring male circumcision is performed by well-trained, adequately equipped and experienced health-care personnel, the complications seen are minor and rare. Table 2 provides a summary of adverse events in all Shang Ring studies conducted on adults in Africa combined (the studies are summarized in Table 1 on page 11).

Table 2: Adverse events in all Shang Ring studies combined. Total number of Shang Ring placements = 1,983		
**Type of event**	**Number**	**Percent (95% CI)**
Serious AEs	0	0.0% (0.0%, 0.2%)
Moderate AEs	20	1.0% (0.6%, 1.6%)
Pain during placement	8	
Pain leading to early removal	2	
Infection	4	
Bleeding	1	
Wound disruption	2	
Insufficient skin removed	3	
Other events	0	
Mild AEs	43	2.2% (1.6%, 2.9%)
Anesthetic complication	1	
Pain during placement	13	
Edema	3	
Injury	3	
Partial detachment	3	
Bleeding	2	
Wound disruption	15	
Insufficient skin removed	3	

Adapted from WHO Technical Advisory Group on Innovations in Male Circumcision: Evaluation of Two Adult Devices. Meeting report: Published in August 2013. Geneva, Switzerland. ISBN 978 92 4 150563 5.

The figures in the above table are for Shang Ring circumcision in adults. In a study in China which involved 824 children the complication included infection (0.4%), edema (3.2%), and insufficient skin removal (0.9%). Average time for wound healing was 13.4 ± 5.8 days after circumcision (Yan, 2010). We found low rates AEs in our recently completed study of use of the Shang Ring in children in Kenya; among the 80 boys under 18 years of age who participated in the study there were no severe AEs and four moderate AEs (three cases of wound disruption, all which healed with conservative management, as well as one case of postoperative pain requiring further analgesics).

During the follow up visits, study staff will assess and provide follow-up care and/or referrals for any post-operative complications. If complications or delayed healing require longer follow-up, men will be followed by study staff as long as necessary.

If clinical care is required after study close-out, care will be provided at the study site as part of their regular activities.

12.3.2 Benefits of the Shang Ring Male Circumcision Procedure

Regardless of the circumcision procedure performed, research studies have shown that circumcised males are at a reduced risk for acquiring urinary tract infections and sexually transmitted infections, including HIV. Circumcision also leads to reduced transmission of human papilloma virus (HPV) and risk of cancer of the cervix in female sexual partners. It also reduces the risk of penile cancer. Circumcision lead to prevention of various conditions affecting the glans and foreskin, including: phimosis,

paraphimosis, balanitis, and posthitis. Circumcised males and their parents/partners report personal hygiene benefits of circumcision.

In addition, data on Shang Ring circumcision in men and boys from Africa (Kenya, Uganda and Zambia) and China suggest that it is associated with easier and simpler circumcision procedure, shorter surgical and recovery times, fewer bleeding complications, and better cosmetic results than standard male circumcision procedures.

12.4 Participant Confidentiality

The confidentiality of all participants enrolled in this research will be protected to the fullest extent possible. Participant's clinic records may be audited by staff from EngenderHealth or Weill Cornell Medical College, regulatory personnel or other individuals authorized to audit the research study. However, participants will not be identified by name on any questionnaire, photograph, electronic case report form (electronic CRF) or any other documentation sent to EngenderHealth or Weill Cornell Medical College or the data management center. Participant identifiers such as names and faces will not be shown on or written on any photograph taken during this research study. All computerized database records will only identify participants by identification numbers.

Participants' study files at the site, including informed consent and electronic case report forms (electronic CRFs), will be kept in a locked file cabinet with access limited to study staff. Participants' information shall be filed by participant identification number (PIN), with names written in a discrete location, i.e. on a page inside the participant's client booklet, in order to reduce the chance of an inadvertent breach of confidentiality. Participants will not be reported by name in any report or publication resulting from data collected in this study.

13.0 DATA MANAGEMENT

13.1 Overview

A detailed data management plan will be written and approved by the PEC before the study begins. Most of the data from the study shall be collected electronically on electronic CRFs (eCRFs) that are loaded on a tablet. The eCRFs will vary somewhat between the two phases of the study.

These data include:

1. Demographic data (Day 0)
2. History and clinical exam findings (Day 0)
3. Screening questions to determine study eligibility (Day 0)
4. Details of the anesthetic procedures and the Shang Ring circumcision (Day 0)
5. Details of the Shang Ring removal and participant's experience while the Shang Ring was in place (Day 7)
6. Participant follow-up data, including occurrence of spontaneous detachment (Day 7,14,21,28,35,42)
7. Information on concomitant medication (all visits)
8. Final interview (In phase I, day complete healing is observed or day 42, whichever comes first. In Phase II on day 42)
9. Details of AEs, SAEs, and SHEs (all visits)

10. Information on protocol violation questionnaire (on occurrence of a protocol deviation or violation)
11. Final status questionnaire

The following data will not be filled/obtained using electronic media.
1. Informed consent and assent forms
2. Participant contact information

13.2 Record Retention and Data Storage

After completion of the study, the EngenderHealth Nairobi and New York offices will retain custody of the data. As most of it will be in electronic format, multiple, secured backup will be made to guard against inadvertent data loss.

The site will keep custody of the originally signed informed consent/assent documents for each participant and all other study documentation for a minimum of 5 years after completion of the study.

For auditing and monitoring purposes or inspections, should the need arise, all data (in printed and electronic media) shall be made available.

13.3 Data Collection, Transmission and Storage

As mentioned, most of the data will be collected on eCRFs. Once a questionnaire is filled, the data will be encrypted on the collecting devices and submitted through a secure connection to the database server. No copy of the information will be left on the electronic device after it has been transferred to the database server. This will ensure that there will be no risk of unauthorized access to the data even in the case of device misplacement. In any case, all devices that will be used to collect data, shall, upon completion of the day's activity be stored under lock and key in the study cabinets in a locked room.

All interactions by the study staff and the collection of data shall require password entry before data collection or transmission takes place. Additionally, password entry will also be required from designated study staff who seek to monitor the study, by viewing the database.

Electronic audit trails will log who and when data was transferred to or accessed on the database ensured more accountable management of the data by the study staff and data management team.

The Data Management Center in collaboration with the Protocol Executive Committee will be responsible for data storage, back-up and retrieval upon request. This way, use of the database will be designated per the role and responsibility of the study/data management staff.

Advantageously, with the use of eCRFs, validation checks shall be used during the data collection process. This way common errors and oversights that take place during data collection shall be eliminated greatly, and thus ensuring "clean" and more useful data is

collected. Hence the security merits and measures that will be used along with electronic data collection include data encryption, use of secure connection for data transfer, not storing a copy of collected data on the device, password-regulated access, audit trail logging and multiple backing-up of study data.

In addition, we may use fingerprint biometrics to accurate identify clients as they come for follow visits. This technology would assist in making sure that participants are correctly identified in the study thus ensuring client confidentiality and accountability of stipends. Some clients may be motivated to misidentify themselves to the study on repeat visits due to perceived benefit e.g. stipend. Clients would be asked to enroll during recruitment whereby biometric information will be taken from two fingers; the right and left index fingers, using a certified fingerprint reader device. This biometric information will be translated into a code containing numbers and letters. No image of the fingerprint will be stored; it will be deleted. The code would be used to identify clients biometrically on repeat visits. It is technically not possible to develop the initial fingerprint image from the code. After study close out all of the biometric information of the clients would be deleted. The institutional review boards shall be informed when this has been done. Much as we would prefer clients to enroll their biometrics for identification during repeat visits, it will NOT be a must for clients to do so should they want to participate in the study.

14.0 QUALITY CONTROL AND QUALITY ASSURANCE

In order to assure quality of the clinical and surgical procedures, the following has or will be done:

- The sites were chosen based on their current knowledge and experience performing conventional male circumcisions in adults, adolescents and younger boys. Additionally, the Homa Bay site has previous experience conducting numerous conventional circumcision outreaches that involve all age groups, one major conventional circumcision study and five clinical studies on the Shang Ring;
- Those male circumcision providers who have not previously used the Shang Ring will be trained locally by EngenderHealth, Cornell and Homa Bay County Hospital staff experienced with use of the Shang Ring;
- All study staff who are not current with research ethics and GCP training will be trained before the start of the study; and,
- The study will be conducted in compliance with the protocol, evolving SOPs, and GCP.

The study sites will maintain all source documents used to complete electronic CRFs, including medical chart notes, laboratory reports, and documentation of referrals, and allow the monitors and other authorized individuals will have access to study documents and pertinent medical records. The study sites may be asked to send copies of some source documentation, with participant identifiers expunged, as well as administrative documentation to EngenderHealth or Weill Cornell Medical College for review.

The investigators and study sites may be subject to a field audit by the sponsor or regulatory agencies to validate the participation of study participants and to verify the

data reported on the eCRFs. This audit could occur while the study is still in progress, several years after the study is completed, or when the data are under review by a regulatory agency. All of the participants' records and other study documentation must be filed and accessible on short notice (three to five working days) during the study and subsequent retention period. Documentation of the study will be adequate for reconstruction of the course of events.

Throughout the study period and after study completion, participant files should be stored in locked file cabinets with access limited to study staff for a minimum of five (5) years.

15.0 SUMMARY OF STUDY MONITORING

During the course of this study, an appropriately trained clinical monitor will maintain regular contact with the study sites and will conduct periodic on-site monitoring visits as needed and as feasible, including visits for training (e.g. research ethics, GCP, study specific), interim monitoring and close-out. The purpose of these visits is to assure that the study is being conducted and informed consent is being obtained according to the approved protocol, GCP, and applicable regulatory guidelines, and to monitor recruitment and data quality.

While some eCRFs may serve as source documents, all documents or records that will serve as source documentation for this study will be fully outlined in the study manual.

Surgical monitoring will be under the purview of Weill Cornell Medical Center and EngenderHealth. Trained surgeons will participate in training activities and regularly monitor the site to assess adherence to acceptable standard of practice and surgical procedures.

16.0 PUBLICATION AND PRESENTATION OF RESULTS

Any and all publications about the study will be developed collaboratively with EngenderHealth, Weill Cornell Medical College and the other study investigators. Furthermore, any presentations/publications about the study results will assign proper credit and acknowledgement to the contributions of the Kenya Ministry of Health, National AIDS/STI Control Programme (NASCOP), Homa Bay County Hospital, Vipingo Health Center, EngenderHealth, and Weill Cornell Medical College.

Subsequently, secondary analysis may be undertaken by either EngenderHealth or Weil Cornell Medical College who have the capability to utilize the data properly and professionally.

No data collected in this study may be published without prior written agreement between EngenderHealth, and Weill Cornell Medical College.

The study will be registered on ClinicalTrials.gov before it begins.

17.0 ACKNOWLEDGMENTS

The PEC thanks the Bill & Melinda Gates Foundation who are funding this study, Renee Rizdon for her guidance and technical insight, and our local partners in Kenya, the Ministry of Health, the National AIDS & STI Control Programme (NASCOP), the Homa Bay County Hospital and Health Management Team (who have facilitated previous studies and trainings on the Shang ring as a device for use in adult male circumcision) and the Kilifi County Health Management team.

18.0 APPENDIXES

Appendix I: Shang Ring circumcision procedure

	Standard Technique	No-flip Technique
1. Measure	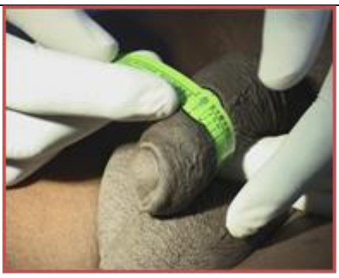	Penis measured to determine Shang Ring size in the same way as for standard technique
2. Place the inner ring	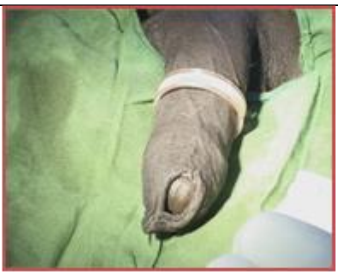 Inner ring placed outside the foreskin	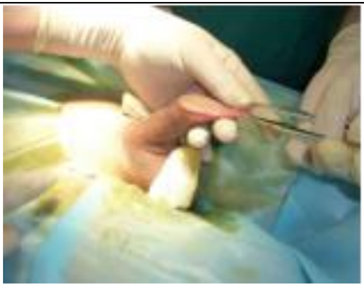 Inner ring placed inside the foreskin
3.Evert the foreskin over the inner ring	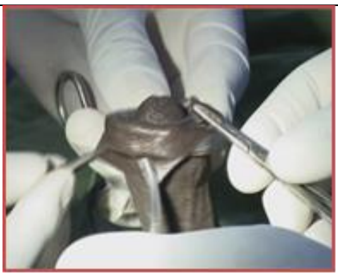	Foreskin does not need to be everted when using the no-flip technique
4. Place the outer ring	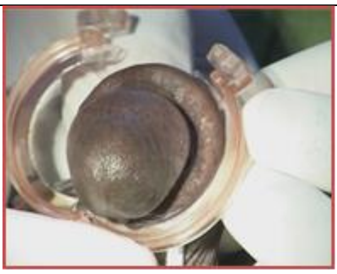	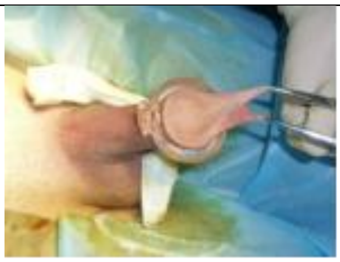
5. Cut off the foreskin	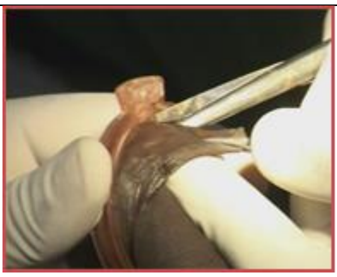	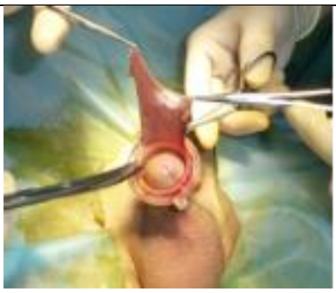

Appendix II: Shang Ring removal procedure

1. Open the inner ring with lock opener	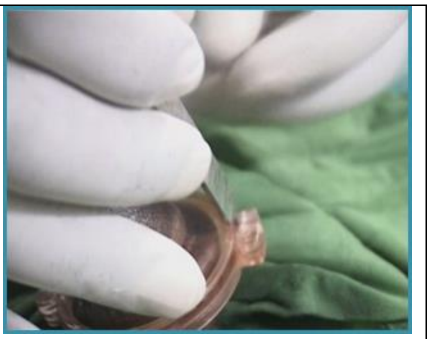
2. Separate dry scub from the inner ring	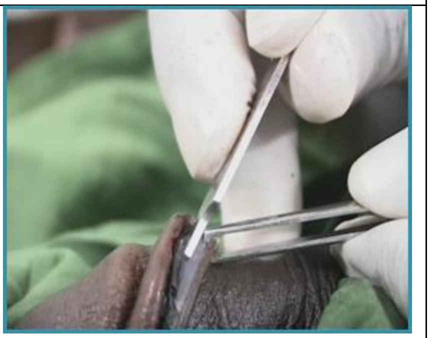
3.Cut off the inner ring	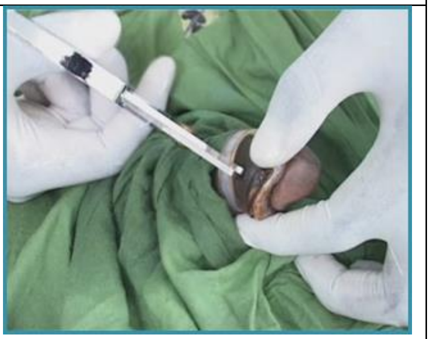
4. Apply bandage around the wound	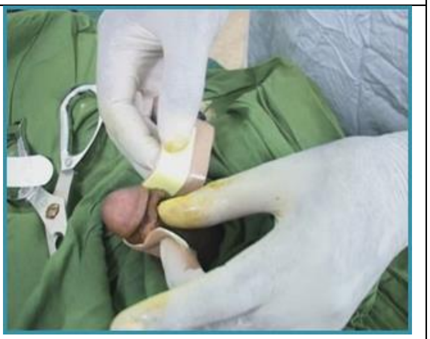

Appendix III: European CE Mark

DNV

DNV BUSINESS ASSURANCE

EC CERTIFICATE - FULL QUALITY ASSURANCE SYSTEM

Certificate No. 4888-2014-CE-RGC-NA
This Certificate consists of 3 pages

This is to certify that the Quality Management System of

Wuhu SNNDA Medical Treatment Appliance Technology Co., Ltd.

P.R. China

for design, production and final product inspection/testing of

Disposable Micro-injury Peritomy Anastomosis Devices

has been assessed with respect to
the conformity assessment procedure described in Article 11.3.a and Annex II excluding section 4 (Module H) of Council Directive 93/42/EEC on Medical Devices, as amended, and found to comply

Further details are given overleaf

Place and date:
Høvik, 01 December 2014

This Certificate is valid until:
01 December 2019

For DNV GL BUSINESS ASSURANCE NORWAY AS

NORWEGIAN ACCREDITATION
PROD 002

CE

Aud Løken Eiklid
Certification Manager

Notified Body No.:
0434

Mariann Jeremiassen
Technical Reviewer

This Certificate has been digitally signed. See www.dnv.com/digitalsignatures for more info

Notice: The certificate is subject to terms and conditions overleaf. Any significant changes in design or construction may render this certificate invalid.

If any person suffers loss or damage which is proved to have been caused by any negligent act or omission of Det Norske Veritas, then Det Norske Veritas shall pay compensation to such person for his proved direct loss or damage. However, the compensation shall not exceed an amount equal to ten times the fee charged for the service in question, provided that the maximum compensation shall never exceed USD 300.000. In this provision "Det Norske Veritas" shall mean the Foundation Det Norske Veritas as well as all its subsidiaries, directors, officers, employees, agents and any other acting on behalf of Det Norske Veritas.

Det Norske Veritas AS, Veritasveien 1, 1322 Høvik, Norway. Tel: +47 67 57 9900 Fax: +47 6757 9911 www.dnv.com
Page 1 of 3

Jurisdiction

Application of Council Directive 93/42/EEC of 14 June 1993, adopted as 'Forskrift for Medisinsk Utstyr' by the Norwegian Ministry of Health and Care Services.

Certificate history

Revision	Description	Issue Date
	Original certificate	**2014-12-01**

Products covered by this Certificate

Product Description	Product	Class
Disposable Micro-injury Peritomy Anastomosis Devices and kits	Self-Locked type, Generation II	IIa
	Round type, Generation III	

The complete list of devices is filed with the Notified Body.

Sites covered by this certificate

Site Name	Address
Wuhu SNNDA Medical Treatment Appliance Technology Co., Ltd.	Overseas Student Pioneer Park, Science Innovation Center, Economic & Technology Zone, Yinhu Road, Wuhu City, P.R. China

EU Representative

Z-Cube Medical Consulting Co., Semmel str.38, 97070 Würzburg, Germany

Terms and conditions

The certificate is subject to the following terms and conditions:

- Any producer (see 2001/95/EC for a precise definition) is liable for damage caused by a defect in his product(s), in accordance with directive 85/374/EEC, as amended, concerning liability of defective products.
- The certificate is only valid for the products and/or manufacturing premises listed above.
- The Manufacturer shall fulfil the obligations arising out of the quality system as approved and uphold it so that it remains adequate and efficient.
- The Manufacturer shall inform the local DNV Office of any intended updating of the quality system and DNV will assess the changes and decide if the certificate remains valid.
- Periodical audits will be held, in order to verify that the Manufacturer maintains and applies the quality system DNV reserves the right, on a spot basis or based on suspicion, to pay unannounced visits.

The following may render this Certificate invalid:

- Changes in the quality system affecting production.
- Periodical audits not held within the allowed time window.

Conformity declaration and marking of product

When meeting with the terms and conditions above, the producer may draw up an EC declaration of conformity and legally affix the CE mark followed by the Notified Body identification number of DNV.

END OF CERTIFICATE

Appendix IV: EN ISO certificate

CERTIFICATE OF REGISTRATION

This is to certify that

Wuhu Snnda Medical Treatment Appliance Technology Co., Ltd.
Science Innovation Centre Economic & Technology Development Zone
4F Overseas Student Pioneer Park, WuHu, AnHui 241007 China

operates a

Quality Management System

which complies with the requirements of

ISO 13485:2003

for the following scope of registration

The registration covers the Quality Management System for design, manufacture and sale of disposable micro-injury peritomy anastomosis device.

Certificate No.: CERT-0061323
File No.: 1628368

Original Date: March 6, 2012
Effective Date: March 6, 2012
Expiry Date: March 5, 2015

Chris Jouppi
President,
QMI-SAI Canada Limited

Guillaume Gignac, ing.f
Vice President, Corporate Operations, Accreditation & Quality
QMI-SAI Canada Limited

Certified System
ISO 13485

SCC Accredited CB-MS
OCSM Accrédité CCN

CMDCAS Recognized Registrar

Registered by:

SAI Global Certification Services Pty Ltd, 286 Sussex Street, Sydney NSW 2000 Australia with SAI Global, 20 Carlson Court, Suite 100, Toronto, Ontario M9W 7K6 Canada. This registration is subject to the SAI Global Terms and Conditions for Certification. While all due care and skill was exercised in carrying out this assessment, SAI Global accepts responsibility only for proven negligence. This certificate remains the property of SAI Global and must be returned to them upon request.
To verify that this certificate is current, please refer to the SAI Global On-Line Certification Register: www.qmi-saiglobal.com/qmi_companies/

SAI GLOBAL
INFORM. INSPIRE. IMPROVE.

DNV BUSINESS ASSURANCE

MANAGEMENT SYSTEM CERTIFICATE

Certificate No. 111795-2012-AQ-RGC-NA

This is to certify that the Management System of:

Wuhu SNNDA Medical Treatment Appliance Technology Co., Ltd.

Overseas Student Pioneer Park, Science Innovation Center, Economic & Technology Zone, Yinhu Road, Wuhu City, P.R. China

has been found to conform to the standard:

ISO 13485:2003

This Certificate is valid for the following product or service ranges:

Design, Manufacture, Sales and Service of Disposable Micro-injury Peritomy Anastomosis Devices.
Supply of Medical Dressings.

Initial Certification date:
18 March 2012

This Certificate is valid until:
18 March 2015

The audit has been performed under the supervision of

Jin Ming Xu
Lead Auditor

NORWEGIAN ACCREDITATION
MSYS 002

Place and date:
Høvik, 18 March 2012

for the Accredited Unit:
DET NORSKE VERITAS CERTIFICATION AS, NORWAY

Eugenie Winger Husebye
Management Representative

Lack of fulfilment of conditions as set out in the Certification Agreement may render this Certificate invalid.

This Certificate has been digitally signed. See www.dnv.com/digitalsignatures for more info

HEAD OFFICE. Det Norske Veritas AS, Veritasveien 1, 1322 Hovik, Norway. Tel: +47 67 57 99 00 Fax: +47 67 57 99 11 - www.dnv.com

19.0 REFERENCES

Albero G, Castellsague X, Giuliano AR, Bosch FX. (2012) Male circumcision and genital human papillomavirus: a systematic review and meta-analysis. Sex Transm Dis. 39(2):104-13.

American Academy of Pediatrics Task Force on Circumcision (2012) Male circumcision. Technical Report. Pedatrics.130(3):e756-85.

Auvert, B., et al. (2005). Randomized, controlled intervention trial of male circumcision for reduction of HIV infection risk: the ANRS 1265 Trial. *PLoS Med, 2*(11). e298.

Auvert, B., et al. (2008). Estimating the resources needed and savings anticipated from roll-out of adult male circumcision in Sub-Saharan Africa. *PLoS ONE, 3*(8), e2679.

Auvert B, Sobngwi-Tambekou J, Cutler E, et al. (2009) Effect of male circumcision on the prevalence of high-risk human papillomavirus in young men: results of a randomized controlled trial conducted in Orange Farm, South Africa. J Infect Dis;199:14.

Backes DM, Bleeker MC, Meijer CJ, Hudgens MG, Agot K, Bailey RC, et al. Male circumcision is associated with a lower prevalence of human papillomavirus-associated penile lesions among Kenyan men. Int J Cancer. 2012 Apr 15;130(8):1888-97.

Bailey R, Moses S, Parker C, et al. (2010) The protective effect of adult male circumcision against HIV acquisition is sustained for at least 54 months: results from the Kisumu, Kenya trial. XVIII International AIDS Conference. Vienna.

Bailey R. C,. Moses, S., Parker, C. B., et al. (2007). Male circumcision for HIV prevention in young men in Kisumu, Kenya: a randomised controlled trial. *Lancet, 369*(9562), 643-56.

Barone MA, Ndede F, Li PS, Masson P, Awori Q, Okech J, Cherutich P, Muraguri N, Perchal P, Lee R, Kim HH, Goldstein M. 2011. The Shang Ring Device for Adult Male Circumcision: A Proof of Concept Study in Kenya. *J Acquir Immune Defic Syndr*. 57:e7–e12

Barone MA, Awori Q, Li PS, Simba RO, Weaver MA, Okech J, Aduda AO, Cherutich P, Muraguri N, Wekesa JM, Nyanchoka J, Perchal P, Masson P, Lee R, Goldstein M, Kioko J, Lusi O, Sokal DC. 2012. Randomized Trial of the Shang Ring for Adult Male Circumcision with Removal at One to Three Weeks: Delayed Removal Leads to Detachment. J Acquir Immune Defic Syndr . 60:e82-e89.

Barone MA et al. (2013). A Field Study of Male Circumcision Using the Shang Ring, a Minimally-Invasive Disposable Device, in Routine Clinical Settings in Kenya and Zambia. 7th International AIDS Society Conference on HIV Pathogenesis, Treatment and Prevention, Kuala Lumpur Malaysia, 30 June-3 July, Abstract No. WELBC04.

Barone MA, Li PS, Awori QD, Lee R, Goldstein M. (2014). Clinical trials using the Shang Ring device for male circumcision in Africa: a review. Special Edition on Male Infertility, Microsurgery and Men's Health, edited by Philip S. Li and Marc Goldstein. Transl Androl Urol. 3(1):113-124.

Bitega JP, Ngeruka ML, Hategekimana T, Asiimwe A, Binagwaho A. (2011). Safety and efficacy of the PrePex device for rapid scale-up of male circumcision for HIV prevention in resource-limited settings. J Acquir Immune Defic Syndr. 15;58(5):e127-34.

Cheng, Y., Peng, Y. F., et al. (2009). A recommendable standard protocol of adult male circumcision with the Chinese Shang Ring: outcomes of 328 cases in China. *Zhonghua Nan Ke Xue 15*(7), 584-92.

DAMOCLES Study Group, NHS Health Technology Assessment Programme. A proposed charter for clinical trial data monitoring committees: helping them to do their job well. The Lancet. 2005; **365**(9460): 711-22.

Davis MA, Gray RH, Grabowski MK, Serwadda D, Kigozi G, Gravitt PE, et al. Male circumcision decreases high-risk human papillomavirus viral load in female partners: a randomized trial in Rakai, Uganda. Int J Cancer. 2013 Sep 1;133(5):1247-52.

Galukande M, Duffy K, Bitega JP, Rackara S, Bbaale DS, Nakaggwa F, Nagaddya T, Wooding N, Dea M, Coutinho A. (2014). Adverse events profile of **PrePex** a non-surgical device for adult male circumcision in a Ugandan urban setting. PLoS One. 28;9(1):e86631.

Gray, R. H., Kigozi G., Serwadda D., et al. (2007). Male circumcision for HIV prevention in men in Rakai, Uganda: a randomised trial*. Lancet, 369*(9562): p. 657-66.

Gray RH, Kigozi G, Serwadda D, Makumbi F, Nalugoda F, Watya S, et al. The effects of male circumcision on female partners' genital tract symptoms and vaginal infections in a randomized trial in Rakai, Uganda. Am J Obstet Gynecol. 2009 Jan;200(1):42 e1-7.

Gray R, Kigozi G, Kong X, Ssempiija V, Makumbi F, Wattya S, et al. The effectiveness of male circumcision for HIV prevention and effects on risk behaviors in a posttrial follow-up study. AIDS. 2012; 26(5): 609-15.

Halperin DT, Bailey RC. Male circumcision and HIV infection: 10 years and counting. Lancet. 1999 Nov 20;354(9192):1813-5.

Kigozi G, Musoke R, Watya S, Kighoma N, Ssebbowa P, Serwadda D, Nalugoda F, Makumbi F, Li P, Lee R, Goldstein M, Wawer M, Sewankambo N, Gray R. J. (2013). The acceptability and safety of the Shang Ring for adult male circumcision in Rakai, Uganda. J Acquir Immune Defic Syndr. 63(5):617-21.

Li HN XJ, Qu LM. (2010). Shang Ring circumcision versus conventional surgical procedures: comparison of clinical effectiveness. Zhonghua Nan Ke Xue. 16(4):325-7.

Lissouba P, Taljaard D, Rech D, Dermaux-Msimang V, Legeai C, Lewis D, Singh B, Puren A, Auvert B. Adult male circumcision as an intervention against HIV: an operational study of uptake in a South African community (ANRS 12126). BMC Infect Dis. 2011 Sep 26;11:253.

Marrazzo JM, Cates W. Interventions to prevent sexually transmitted infections, including HIV infection. Clin Infect Dis. 2011 Dec;53 Suppl 3:S64-78.

Ministry of Public Health and Sanitation (MOPHS) [Kenya]. Kenya national strategy for voluntary medical male circumcision. Nairobi: MOPHS; 2009. Available from: http://nascop.or.ke/library/VMMC/VMMC%20Strategy.pdf

Mutabazi V, Kaplan SA, Rwamasirabo E, Bitega JP, Ngeruka ML, Savio D, Karema C, Binagwaho A. (2013). One-arm, open-label, prospective, cohort field study to assess the safety and efficacy of the PrePex device for scale-up of nonsurgical circumcision when performed by nurses in resource-limited settings for HIV prevention. J Acquir Immune Defic Syndr. 1;63(3):315-22.

Nasio JM, Nagelkerke NJ, Mwatha A, Moses S, Ndinya-Achola JO, Plummer FA. Genital ulcer disease among STD clinic attenders in Nairobi: association with HIV-1 and circumcision status. Int J STD AIDS. 1996 Oct;7(6):410-4.

Pan F, Pan L, Zhang A, Liu Y, Zhang F, Dai Y. (2013). Circumcision with a novel disposable device in Chinese children: A randomized controlled trial. Int J Urol;20(2):220-6.

Peng, Y. F., et al. (2008). Clinical application of a new device for minimally invasive circumcision*.* *Asian J Androl, 10*(3), 447-54.

Serwadda D, Wawer MJ, Makumbi F, Kong X, Kigozi G, Gravitt P, et al. Circumcision of HIV-infected men: effects on high-risk human papillomavirus infections in a randomized trial in Rakai, Uganda. J Infect Dis. 2010 May 15;201(10):1463-9.

Sokal DC, Li PS, Zulu R, Awori QD, Combes SL, Simba RO, Lee R, Hart C, Perchal P, Hawry HJ, Bowa K, Goldstein M, Barone MA. 2014. Randomized Controlled Trial of the Shang Ring Versus Conventional Surgical Techniques for Adult Male Circumcision: Safety and Acceptability. JAIDS. 65: 447–455.

Taddio A. 2001. Pain management for neonatal circumcision. Paediatr Drugs.;3(2):101-11.

Wawer MJ, Tobian AA, Kigozi G, Kong X, Gravitt PE, Serwadda D, et al. Effect of circumcision of HIV-negative men on transmission of human papillomavirus to HIV-negative women: a randomised trial in Rakai, Uganda. Lancet. 2011 Jan 15;377(9761):209-18.

Wilson LE, Gravitt P, Tobian AA, Kigozi G, Serwadda D, Nalugoda F, et al. Male circumcision reduces penile high-risk human papillomavirus viral load in a randomised clinical trial in Rakai, Uganda. Sex Transm Infect. 2013 May;89(3):262-6

WHO/UNAIDS. (2007) New Data on Male Circumcision and HIV Prevention: Policy and Programme Implications. WHO/UNAIDS Technical Consultation on Male Circumcision and HIV Prevention: Research Implications for Policy and Programming. Montreux: Conclusions and Recommendations; March 6–8, 2007. Geneva: WHO.

World Health Organization, UNAIDS, and JHPIEGO. (2008). *WHO Manual for Male Circumcision under Local Anesthesia*. Geneva, Version 2.5c.

World Health Organization. (2012) Framework for Clinical Evaluation of Devices for Male Circumcision. Geneva. September 2012.

World Health Organization. (2013a) Progress in scaling up voluntary medical male circumcision for HIV prevention in East and Southern Africa, January – December 2012. Republic of Congo: WHO Regional Office for Africa.

WHO Technical Advisory Group on Innovations in Male Circumcision: Evaluation of Two Adult Devices. (2013b) Meeting report. Geneva, Switzerland. http://apps.who.int/iris/bitstream/10665/85269/1/9789241505635_eng.pdf

Yan B, You H, Zhang K, Tang HY, Mao W, He GH, Yin ZG. (2010). Circumcision with the Chinese Shang ring in Children: Outcome in 824 cases. Chinese National Journal of Andrology, 2010, 16(3): 250-253
